# A comparative study of synthetic winged peptides for absolute protein quantification

**DOI:** 10.1038/s41598-021-90087-9

**Published:** 2021-05-25

**Authors:** Eliska Benesova, Veronika Vidova, Zdenek Spacil

**Affiliations:** Faculty of Science, Masaryk University, RECETOX, Kamenice 753/5, Pavilion D29, 625 00 Brno, Czech Republic

**Keywords:** Peptides, Proteins, Proteolysis, Proteomics

## Abstract

A proper internal standard choice is critical for accurate, precise, and reproducible mass spectrometry-based proteomics assays. Synthetic isotopically labeled (SIL) proteins are currently considered the gold standard. However, they are costly and challenging to obtain. An alternative approach uses SIL peptides or SIL "winged" peptides extended at C- or/and N-terminus with an amino acid sequence or a tag cleaved during enzymatic proteolysis. However, a consensus on the design of a winged peptide for absolute quantification is missing. In this study, we used human serum albumin as a model system to compare the quantitative performance of reference SIL protein with four different designs of SIL winged peptides: (i) commercially available SIL peptides with a proprietary trypsin cleavable tag at C-terminus, (ii) SIL peptides extended with five amino acid residues at C-terminus, (iii) SIL peptides extended with three and (iv) with five amino acid residues at both C- and N-termini. Our results demonstrate properties of various SIL extended peptides designs, e.g., water solubility and efficiency of trypsin enzymatic cleavage with primary influence on quantitative performance. SIL winged peptides extended with three amino acids at both C- and N-termini demonstrated optimal quantitative performance, equivalent to the SIL protein.

## Introduction

The advent of mass spectrometry-based proteomics allowed for accurate quantification of a panel of target proteins^1^ in complex biological samples with high selectivity, sensitivity, and multiplexing capacity^2,3^. A typical "bottom-up" workflow applies enzymatic proteolysis to generate tryptic peptides as surrogates of target proteins^4^. Proteotypic peptides are quantified using liquid chromatography (LC) or ultra-high performance LC (UHPLC) separation techniques coupled with mass spectrometry (MS) in selected reaction monitoring (SRM) detection mode^5^.

Absolute quantification of a target protein by MS technique requires an internal standard, typically a synthetic isotopically labeled (SIL) proteotypic peptide or a recombinant protein^6^. SIL analogs of surrogate peptides ("heavy peptides") are added to a sample in a known concentration and used to calculate the concentration of analyte ("light peptide") in the sample from the light-to-heavy ratio. SIL peptide selection or protein selection is critical for robust, accurate, and precise protein assay^1,7^. SIL proteins are considered the gold standard with reportedly optimal quantification performance^1^. SIL protein properties are identical with the corresponding target protein and efficiently mitigate the inherent variance, particularly the incomplete enzymatic proteolysis. However, the availability of recombinant SIL proteins is limited.

On the other hand, SIL analogs of tryptic proteotypic peptides are readily available. Tryptic SIL peptides are typically added to a sample after enzymatic digestion to correct for biases associated with solid-phase extraction (SPE) (i.e., peptide recovery) and LC–MS analysis (i.e., ionization efficiency, relative response, and matrix effects)^1,8^. The internal standardization with tryptic SIL peptides fails to normalize for the enzymatic digestion variance, the major contributor to the irreproducibility in a "bottom-up" proteomics protocol^9–11^. On the other hand, tryptic SIL peptides extended at the C-terminus or both C- and N-termini with the natural sequence of amino acids corresponding to a target protein or artificial trypsin cleavable tag (TCT) efficiently normalize the variability in proteolysis. Literature refers to extended peptides as "winged," "elongated," or "cleavable" and describes a varied length of extension (i.e., 2–7 amino acids) positioned solely at C-terminus or both C- and N-termini^3,12–21^. Studies only focusing on N-terminal extended peptides are not available.

Studies focused on SIL proteins' quantitative performance relative to tryptic SIL, and SIL-extended (SIL-Ex) peptides report ambiguous and controversial results. Some did not observe SIL-Ex peptides' advantages over tryptic SIL peptides regarding the precision and accuracy of a protein assay^3,22^. Others reported improved quantitative performance of SIL-Ex peptides in comparison to tryptic SIL peptides^11,13^. However, a comprehensive comparison of SIL-Ex peptides, tryptic SIL peptides, and SIL protein quantitative performance was not performed. For instance, Scott et al. compared SIL extended peptides with two, four, and six amino acids at both C- and N-termini and attributed the superior quantitative performance to six amino acid extension^11^. However, the influence of the sequence extension position (C- or N-terminus) requires further investigation as the body of published studies is limited.

**Figure 1:**
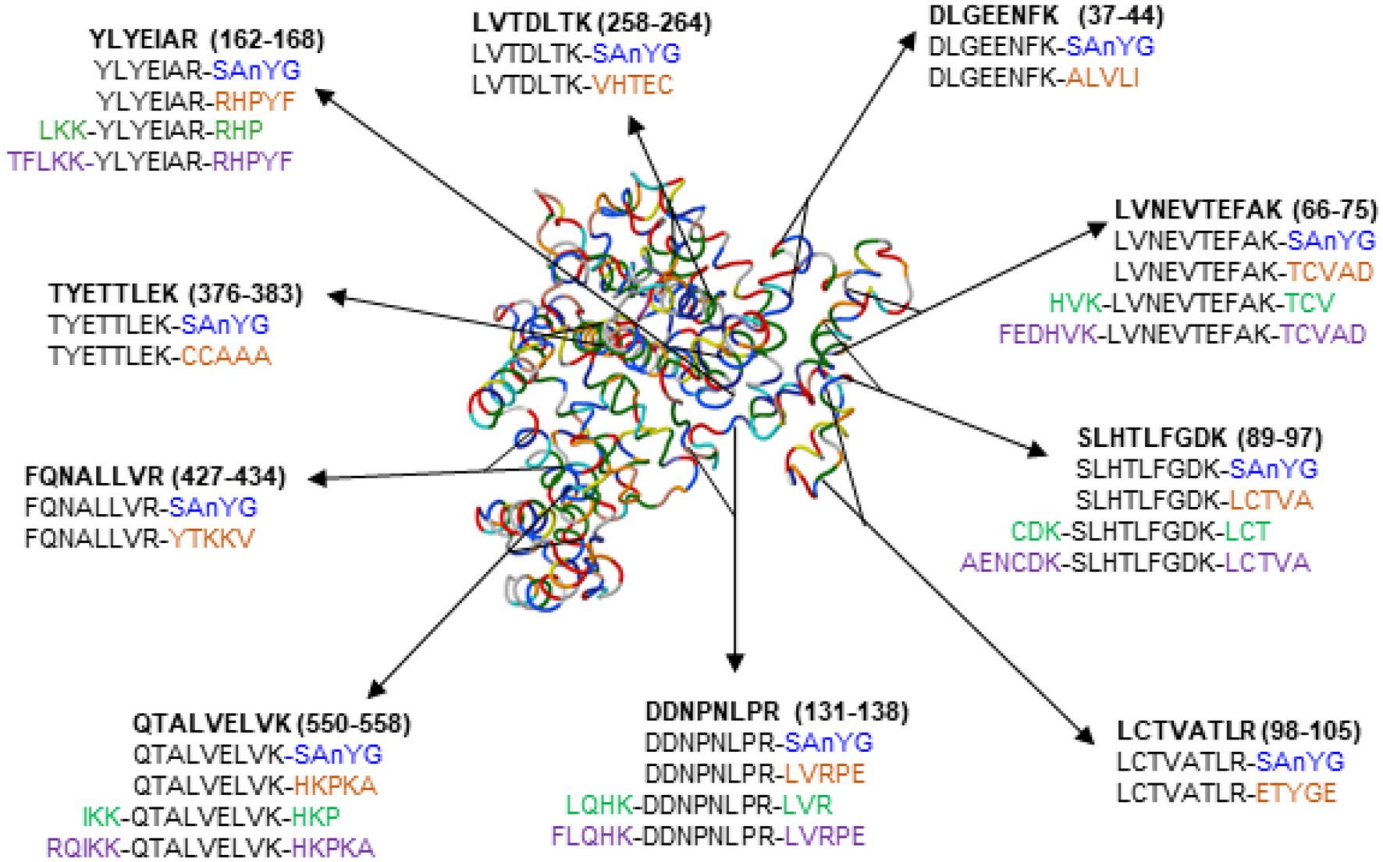
Signature proteotypic peptides are marked in bold, a peptide position in the HSA protein sequence in brackets. Compared types of SIL-Ex peptides are shown in different colors: SIL-TCT (blue), SIL-ExC5 (orange), SIL-ExC3N3 (green), SIL-ExC5N5 (purple). SIL-ExC5, SIL-ExC3N3, and SIL-ExC5N5 denote signature peptides extended with the natural sequence of amino acids corresponding to the target protein sequence. SIL-TCT represent signature peptides extended with a tetrapeptide (SAnYG, nY = nitrotyrosine) tag.

In this study, we elucidate the influence of the length and position of a sequence extension on quantitative performance in the human serum albumin (HSA) model system. Our results demonstrate the dependence of enzymatic digestion efficiency, solubility, and quantitative performance on the sequence extension design. We tested the quantitative performance of (i) commercially available SIL-TCT peptides extended with a tetrapeptide (serine-alanine-nitrotyrosine-glycine) tag at C-terminus, (ii) SIL-Ex peptides with five amino acids at C-terminus (SIL-ExC5), (iii) SIL-Ex with three amino acids at both C- and N-termini (SIL-ExC3N3), and (iv) SIL-Ex with five amino acids at both C- and N-termini (SIL-ExC5N5) corresponding to target protein sequence (Fig. 1). We compared SIL-TCT and SIL-Ex peptides' quantitative performance to a gold standard SIL-HSA protein as a reference method.

## Methods

### Chemicals and solvents

Formic acid (FA) for mass spectrometry (cat. #94318), sodium deoxycholate (SDC) BioXtra ≥ 98.0% (cat. #30970), ammonium bicarbonate (AmBic) BioUltra ≥ 99.5% (cat. #09830), and iodoacetamide (IAA) ≥ 99.0% (cat. #I6125) were all purchased from Sigma Aldrich (St. Louis, MO); 1,4-dithiothreitol (DTT) ≥ 99.0% (cat. #6908, Carl Roth, Karlsruhe, Germany); Trypsin Gold, Mass spectrometry grade (cat. #V5280, Promega, Madison, WI); LC–MS grade acetonitrile (ACN, cat. #1207802BS, Biosolve, Valkenswaard, The Netherlands). Water was produced using Millipore Simplicity 185 ultrapure water system (Merck Millipore corp. Billerica, MA).

### Synthetic isotopically labeled peptides and recombinant HSA protein

SIL peptides with C-terminal arginine (R*, ^13^C_6_H_14_O_2_^15^N_4_) or lysine (K*, ^13^C_6_H_14_O_2_^15^N_2_) extended with (i) a tetrapeptide SAnYG tag (SpikeTides_TQL™), (ii) five amino acids at C-terminal R*/K* (SIL-ExC5), (iii) three amino acids at both C- and N-termini (SIL-ExC3N3) or iv) five amino acids at both C- and N-termini with (SIL-ExC5N5) were custom-ordered from JPT Technologies (Berlin, Germany). SIL peptides were synthesized with alkylated cysteines. Sequences of SIL-Ex peptides, molecular weights, and quantifier transitions are shown in Table S1. The purity of all peptides (> 95%) was determined using RP-HPLC–UV (220 nm, C18, linear gradient) and the absolute concentration by amino acid analysis. Each peptide acronyms include the sequence of the first four N-terminal amino acids. The recombinant SIL-HSA protein (> 98%, cat. #MSST0011, Sigma Aldrich) was reconstituted in 5% ACN to prepare a stock solution (500 nM).

### Human serum samples

Individual human serum samples were collected during a one-time morning session from 14 healthy adult volunteers (seven women and seven men). Venous blood collected into 9 mL serum tubes was allowed to clot and centrifuged (10 min, 2500×*g*, 20 °C). Individual serum samples were pooled and stored at − 20 °C until analysis. Signed informed consent forms were obtained from all participants and archived. The study was approved by the Committee for Ethics of CELSPAC: TNG (CELSPAC/EK/4/2016) at University Hospital Brno, Czech Republic, under the Declaration of Helsinki. The methods used in the study and described below were carried out following the relevant guidelines and regulations. The authors confirm that the data supporting the findings of this study are available within the article and in supplementary materials.

### In-solution proteolysis by trypsin

HSA concentration in the pooled serum sample diluted twofold with ultrapure water was determined using BCG albumin assay kit on the 96-well plate format (cat. #MAK124-1KT, Sigma-Aldrich). The seven-point calibration series was prepared using HSA standard (5 to 50 mg/mL) in ultrapure water. The absorption spectrophotometry was measured at 620 nm wavelength. The pooled serum sample (10 µL), diluted 2000-fold using 50 mM AmBic with 5 mg/mL SDC (AmBic/SDC buffer) to HSA concentration of approx. 500 nM and transferred into a clean microcentrifuge tube. After adding SIL protein or peptide internal standard (10 µL), the sample was reduced (adding 1 µL of 200 mM DTT stock solution, 95 °C, 10 min), cooled down to ambient temperature, and cysteine residues were alkylated (adding 1 µL of 400 mM IAAstock solution, ambient temperature, 30 min in the dark). Trypsin was added to the sample in a 1:20 ratio (w/w enzyme to total protein content), and samples were incubated (37 °C, 16 h) in microcentrifuge tubes sealed with a paraffin film. The enzymatic proteolysis was quenched at different time points (0.5, 2, 4, 8, 16, and 24 h; n = 3) to optimize the incubation time. The proteolysis was quenched by adding 200 µL of 2% FA in water (pH < 3), and tryptic peptides were desalted using a mixed-mode SPE cartridge in a 96-well plate format (Oasis PRIME HLB, Waters, Milford, MA). Samples were loaded onto the SPE cartridge, washed (300 µL water with 2% FA, pH < 3), eluted (50% ACN; 2% FA in water, pH < 3), and the extract was dried in a vacuum concentrator centrifuge (Savant SPD121 P SpeedVac, Thermo Fisher). Samples were dissolved (10 µL) in 5% ACN with 0.1% FA in water before UHPLC-SRM analysis.

### Liquid chromatography and mass spectrometry protein assays

Samples were analyzed using the UHPLC system (Infinity™ 1260 Agilent Technologies, Santa Clara, CA) and a reversed-phase analytical column (C_18_ Peptide CSH; 1.7 µm, 2.1 mm i.d. × 100 mm; cat. #186006937; Waters; Milford, MA). The column and the autosampler temperatures were 40 °C and 8 °C, respectively, and the sample injection volume was 3 µL. The mobile phase flow rate was 0.3 mL/min using mobile phase A (0.1% FA in water) and B (0.1% FA in 95% ACN) in the linear gradient elution mode (0–15 min) with a wash step (15.30–20 min) and re-equilibration step (21–25 min). The gradient program was: 0.0 min 5% B; 15 min 20% B; 15.3 min 95% B; 20 min 95% B; 21 min 5% B; 25 min 5% B. A triple quadrupole mass spectrometer (6495B, Agilent Technologies, USA) was used for SRM assays in positive ion mode. A standard-flow Jet Stream electrospray ionization (ESI) source parameters were: capillary voltage 3.5 kV, gas flow rate 18 L/min at 220 °C, sheath gas pressure 25 PSI and flow rate 12 L/min at 400 °C and nozzle voltage 800 V. The acquisition in dynamic SRM mode was centered on the peptide experimental retention time within 3 min window. All tryptic peptides and SIL peptides internal standards were analyzed using 3–5 SRM qualifiers and one quantifier SRM transition. In total, 96 transitions were monitored (Table S2) with a total cycle time of 500 ms. SRM peak areas were reported in Skyline (ver. 20.1.0.155, MacCoss Lab., UW, USA) and MassHunter (Agilent Technologies). Data were processed further in Excel (MS Office Professional Plus, 2013), and the statistical analysis and data evaluation performed in GraphPad PRISM (ver. 8.0.2).

### Design of SRM protein assays

SRM Atlas (www.srmatlas.org) and Skyline software guided the selection of tryptic proteotypic peptides with sequence length restriction to 7–20 AA. In total, 27 peptides were selected, 13 peptides (65 transitions) via SRM atlas (Table S3), and 24 peptides (75 transitions) via Skyline (Table S4). Ten signature peptides overlapped between SRM Atlas and Skyline (Table S5). UHPLC-SRM screening in a 100-fold diluted pooled serum sample was performed to select surrogate peptides based on relative intensities/peak areas, peak shapes, and the HSA protein sequence comprehensive coverage. The proteotypic amino acid sequences of signature peptides selected for HSA were verified using a BLAST search of the Homo sapiens genome in the NextProt database.

### The solubility of synthetic peptides

The hydrophobicity indexes and predicted water solubility of SIL peptides are in Table S6. Hydrophobicity indexes were calculated using Thermo Fisher Peptide analyzing tool^23^. The Innovagen Peptide solubility calculator was used to predict water solubility^24^. Lyophilized custom-ordered peptides were reconstituted in three different buffers: (i) 5% ACN in AmBic/SDC, (ii) 20% ACN in AmBic/SDC, and (iii) 50% ACN in AmBic/SDC. Stock solutions (10 µM) were prepared for each individual SIL-TCT and SIL-Ex peptide. The equimolar concentration (500 nM) mixed working solutions containing all peptides were prepared for SIL-TCT peptides and each SIL-Ex peptide design. The working solutions of SIL peptides (10 µL) were added to a 2000-fold diluted serum sample. Triplicate samples of all SIL peptide designs were prepared in each solvent system, processed with proteolytic protocol, and analyzed by UHPLC-SRM. The HSA sample concentration (nM) was determined using the SIL peptides of each design. We tested for the agreement between the experimental and the actual HSA concentration determined by reference methods to select an optimal solvent system.

## Results and discussion

### Design of quantitative protein assay

The selection of optimal signature peptides as protein surrogates is an essential step to develop selective, accurate, and precise protein assay^5^. Signature peptides have to be chemically stable, detectable with UHPLC-MS, and proteotypic, i.e., specific to the target protein. For this comparative study, we selected HSA protein, the principal constituent of blood plasma (55–60%)^25,26^, as a model system. Globular HSA protein consists of 609 amino acids (MW 66.5 kDa), forming a signal sequence (1–18 AA), a propeptide (19–24 AA), and a native mature protein (25–609 AA)^27^. We selected the initial list of 27 signature peptide candidates (10 in common for SRMAtlas and Skyline, three unique for SRM, and 14 unique for Skyline. The peptide location in the HSA protein sequence, quantifier SRM precursor and product ions, experimental retention times, and integrated peak areas from the initial screening are listed in Table S5. Four peptides selected under Skyline software's guidance (CCAAADPHECYAK, VHTECCHGDLLECADDR, RPCFSALEVDETYVPK, ETCFAEEGK) were not detected in the initial screening step. The best performing surrogate peptides (10) were selected based on the UHPLC-MS screening (i.e., high relative response/peak area, the symmetric chromatographic peak) to cover the HSA protein sequence uniformly. One selected peptide (LCTV) contained cysteine residue. However, the sample preparation protocol uses reduction and alkylation steps to eliminate cysteine residue's influence on quantitative performance. SIL peptides were synthesized with alkylated cysteine. The SRM library for both SIL peptide and light serum peptide LCTV included cysteine alkylation (+ 57 Da). Typical chromatograms of candidate peptides with highlighted best performing surrogate peptides (10) are shown in Fig. S1.

### The quantitative performance of C-terminal extended synthetic peptides

**Figure 2:**
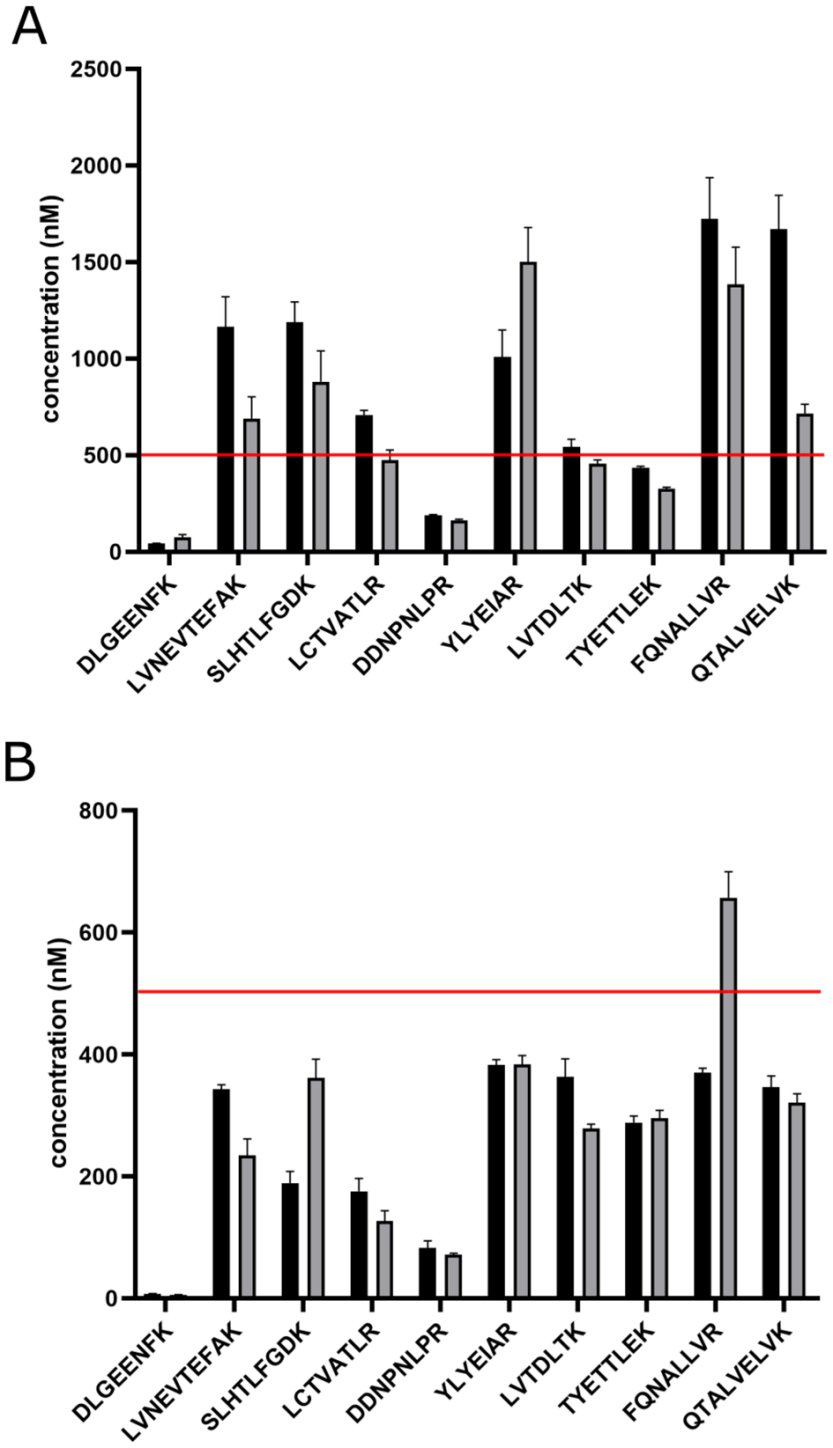
The quantitative performance depends on the reconstitution solvent for SIL-TCT (black) and SIL-ExC5 (grey) peptides. The reconstitution solvent was water with 5% ACN (**A**) and AmBic/SDC buffer with 5% ACN (**B**). The reference HSA protein concentration marked with the red line at 500 nM (determined by BCG assay and protein assay using SIL-HSA).

The best-performing surrogate peptides (10) were selected to cover the HSA protein sequence (Fig. 1). The SIL peptides extended with (i) commercial tetrapeptide (SAnYG) trypsin cleavable tag (TCT) and (ii) five amino acids at C-terminal R*/K* (ExC5) were compared. The water solubility and hydrophobicity scores were calculated (Table S6), except for hydrophobicity of SIL-TCT peptides containing nitrotyrosine modification. Three SIL-TCT sequences (i.e., YLYE, LCTV, FQNA) indicated low water solubility. SIL-ExC5 peptides, except for YLYE and SLHT, exhibited moderate hydrophobicity and good water solubility. We compared the quantitative performance using 5% ACN and AmBic/SDC buffer with 5% ACN to dissolve lyophilized SIL peptides. We initially tested SIL-TCT and SIL-ExC5 peptides reconstitution in water with 5% ACN, a solvent compatible with trypsin digestion and UHPLC-MS analysis. SIL peptides in water with 5% ACN were added into the sample diluted with AmBic/SDC buffer for pH optimal during enzymatic proteolysis. We calculated the concentration of light HSA peptides based on the light-to-heavy ratio. Average HSA concentrations determined by SIL-ExC5 and SIL-TCT peptides initially reconstituted in water with 5% ACN were falsely high at 666.73 nM and 867.70 nM, respectively, compared to the actual concentration determined by reference BCG assay and protein assay internally standardized with SIL-HSA (Fig. 2A and Table S7). HSA protein concentrations reported by individual SIL-ExC5 and SIL-TCT peptides ranged between 74.88 nM to 1,501.44 nM and 42.53 nM to 1,723.73 nM, respectively (Fig. 2A and Table S7). The HSA concentration was accurately (< 20% CV) determined by only two SIL-ExC5 peptides (i.e., LVTD and LCTV) and two SIL-TCT peptides (i.e., TYET and LVTD). Falsely low HSA protein concentrations reported by SIL-TCT and SIL-ExC5 peptides DDNP and DLGE were perhaps due to higher proteolytic SIL peptide yields relative to corresponding light HSA peptides. On the contrary, falsely high HSA protein levels were determined by five SIL-TCT and SIL-ExC5 peptides (i.e., SLHT, YLYE, FQNA, LVNE, QTAL). In this case, the inaccurate HSA determination is probably due to SIL peptides' low solubility in reconstitution solvent (5% ACN in water). The lower SIL peptide concentration resulted in a higher ratio of the SRM peak area of target HSA peptide (light) to SIL peptide internal standard peak area (heavy). All five peptides indicated high hydrophobicity indexes, and limited water solubility was predicted for SLHT, YLYE, and FQNA (Table S6).

Peptide solubility improves with adding dimethylformamide, dimethyl sulfoxide, acetic acid, detergents, or increasing organic solvent content (i.e., ACN, MeOH). However, dimethylformamide, dimethyl sulfoxide, and detergents are not compatible with the MS technique, and high organic content reduces the trypsin digestion efficiency^28^. We improved the solubility of lyophilized SIL-ExC5 and SIL-TCT surrogate peptides, reconstituting them in AmBic/SDC buffer with 5% ACN. A bile salt surfactant SDC is compatible with trypsin digestion workflow up to 1% w/v^29^ and removable before UHPLC-MS analysis by acidic precipitation^30^. AmBic/SDC buffer was demonstrated to improve trypsin digestion efficiency and reproducibility over other additives (e.g., urea or guanidine hydrochloride)^30^. However, the average HSA concentration (n = 10) quantified by SIL-ExC5 and SIL-TCT peptides initially reconstituted in AmBic/SDC buffer with 5% ACN was falsely low 273.41 nM and 254.69 nM, respectively, (Table S7). HSA concentrations determined by individual SIL-ExC5 and SIL-TCT peptides ranged from 5.42 to 656.61 nM and from 7.40 to 382.39 nM, respectively (Fig. 2B and Table S7). None of the SIL-ExC5 or SIL-TCT peptides accurately determined the HSA protein concentration (< 20% CV). All reported concentrations were falsely low (Fig. 2 and Table S7).

In summary, the quantitative performance of SIL-ExC5 and SIL-TCT peptides was insufficient. SIL-ExC5 and SIL-TCT peptides DDNP and DLGE reported falsely low HSA concentration in both 5% ACN in water and 5% ACN in AmBic/SDC reconstitution buffers, probably due to tryptic yields different from light peptides. Peptides LVNE, SLHT, YLYE, and QTAL reported highly discordant HSA concentrations between SIL-TCT and SIL-ExC5 peptides and reconstitution solvents (i.e., water with 5% ACN and AmBic/SDC buffer with 5% ACN) summarized in Fig. 2 and Table S7. To investigate the nature of quantitative performance issues, we obtained SIL versions extended at both C- and N-termini with three (ExC3N3) and five (ExC5N5) amino acids for problematic surrogate peptides (i.e., LVNE, SLHT, DDNP, YLYE, QTAL).

### Optimal incubation buffer for extended synthetic peptides

Hydrophobicity and water solubility are proportional to the peptide length and primary structure. We calculated the hydrophobicity index for signature peptides, and all tested SIL-Ex peptides, except for SIL-TCT peptides modified with nitrotyrosine (Table S6)^23^. Expectedly, hydrophobicity indexes were the highest for SIL-ExC5N5, with an average value of 35.6. In comparison, an average hydrophobicity index for selected tryptic signature peptides was 20.6, for SIL-ExC5 and SIL-ExC3N3 peptides were 28.1 and 28.8, respectively.

**Figure 3:**
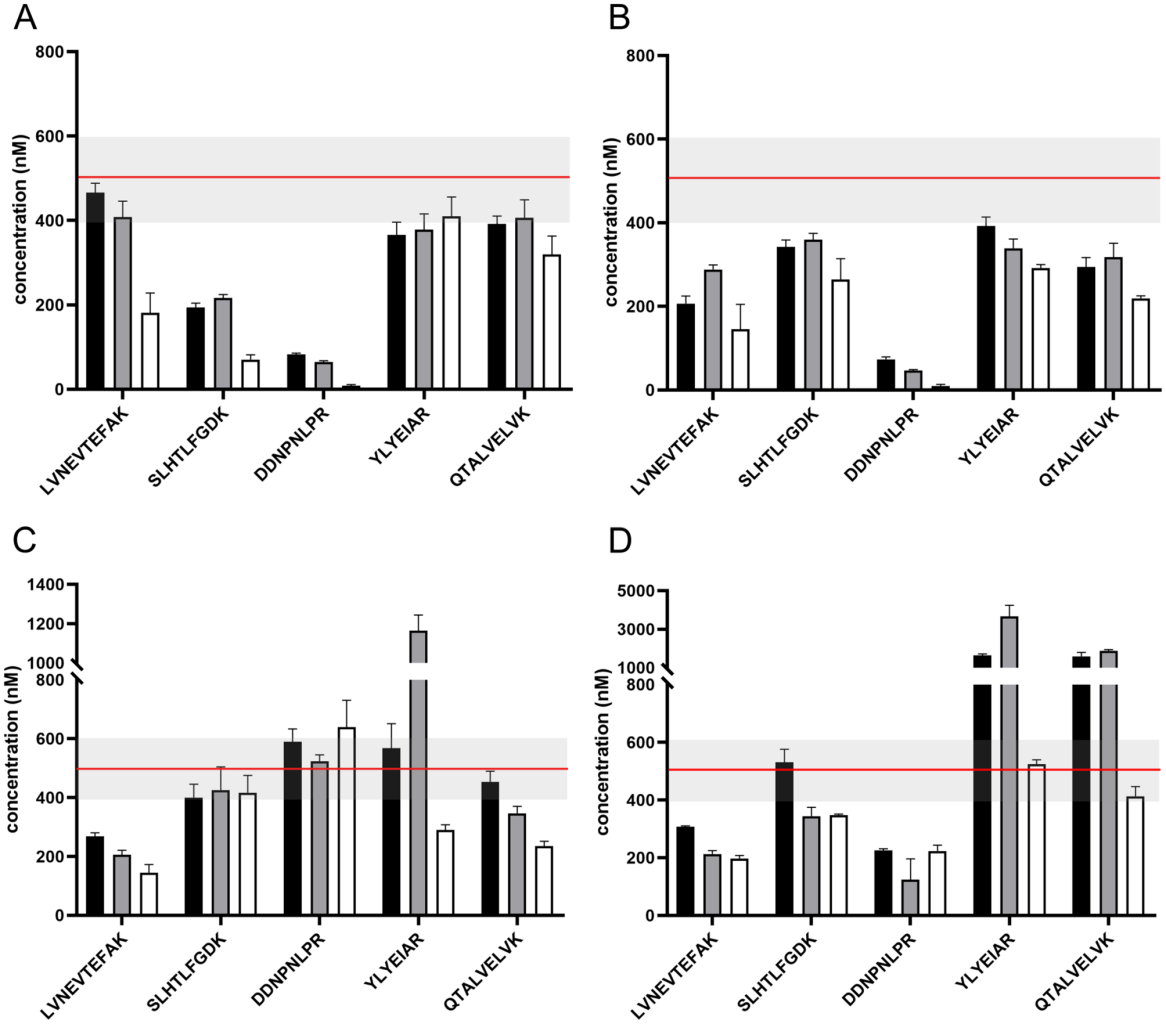
The effect of organic content in the reconstitution solvent on the quantitative performance of SIL-Ex peptides. HSA protein concentration was determined using SIL-TCT (**A**), SIL-ExC5 (**B**), SIL-ExC3N3 (**C**), and SIL-ExC5N5 (**D**) peptides reconstituted in AmBic/SDC buffer with 5% (black), 20% (grey), and 50% ACN (white). HSA concentration determined by reference methods (500 nM) indicated as a red line; the grey area represents 20% tolerance.

To simultaneously optimize the solubility and effects of high organic content on proteolysis, we reconstituted each lyophilized SIL-Ex peptide in AmBic/SDC buffer with i) 5%, ii) 20%, and iii) 50% of ACN. We added the mixed solution of SIL-Ex peptides (10 µL) to the pooled serum sample (10 µL) diluted using AmBic/SDC buffer, resulting in 2.5%, 10%, and 25% of ACN in the incubation buffer, respectively. We measured SRM peak areas of signature peptides from light HSA and SIL-Ex peptides. Tryptic peptide yields are reported relative to light HSA signature peptides incubated in AmBic/SDC buffer with 2.5% ACN. As expected, the efficiency of the trypsin digestion of HSA protein decreased with an increasing percentage of ACN in the proteolytic buffer (Fig. S2). In particular, SRM peak areas of DDNP and SLHT peptides were affected (approx. tenfold decrease, Fig. S2). Perhaps a consequence of limited accessibility of hydrophilic locations in light HSA protein (partial loss of solubility) or slower interaction with trypsin active site (conformation change). Interestingly, the SRM peak areas of SIL-ExC5 and SIL-TCT peptide DDNP remain constant, suggesting that primarily N-terminal cleavage's efficiency is affected by the high ACN content. Tryptic yields of hydrophobic signature peptides YLYE, LVNE, and QTAL were less sensitive to the ACN in the proteolytic buffer. The yields of tryptic signature peptides YLYE, LVNE, and QTAL formed from light HSA were highly discordant with SIL-ExC5N5 peptides' yields, probably due to different solubility of HSA protein and SIL-ExC5N5 peptides. The quantitative performance of SIL-ExC5N5 peptides with high hydrophobicity indexes (i.e., YLYE and QTAL) was optimal when reconstituted in AmBic/SDC buffer with 50% ACN (Fig. 3D). Yields of signature peptides YLYE and QTAL formed from SIL-ExC5N5 were up to threefold higher when reconstituted in AmBic/SDC buffer with 50% ACN compared to 5% ACN content (Fig. S2). However, the high content of ACN during proteolysis unfavorably reduced tryptic peptide yields from HSA protein. The proteolytic buffer with minimal ACN content was optimal for SIL-TCT, SIL-ExC5, and SIL-ExC3N3 peptides and thus selected for further investigations (Fig. 3).

### The enzymatic digestion efficiency

**Figure 4:**
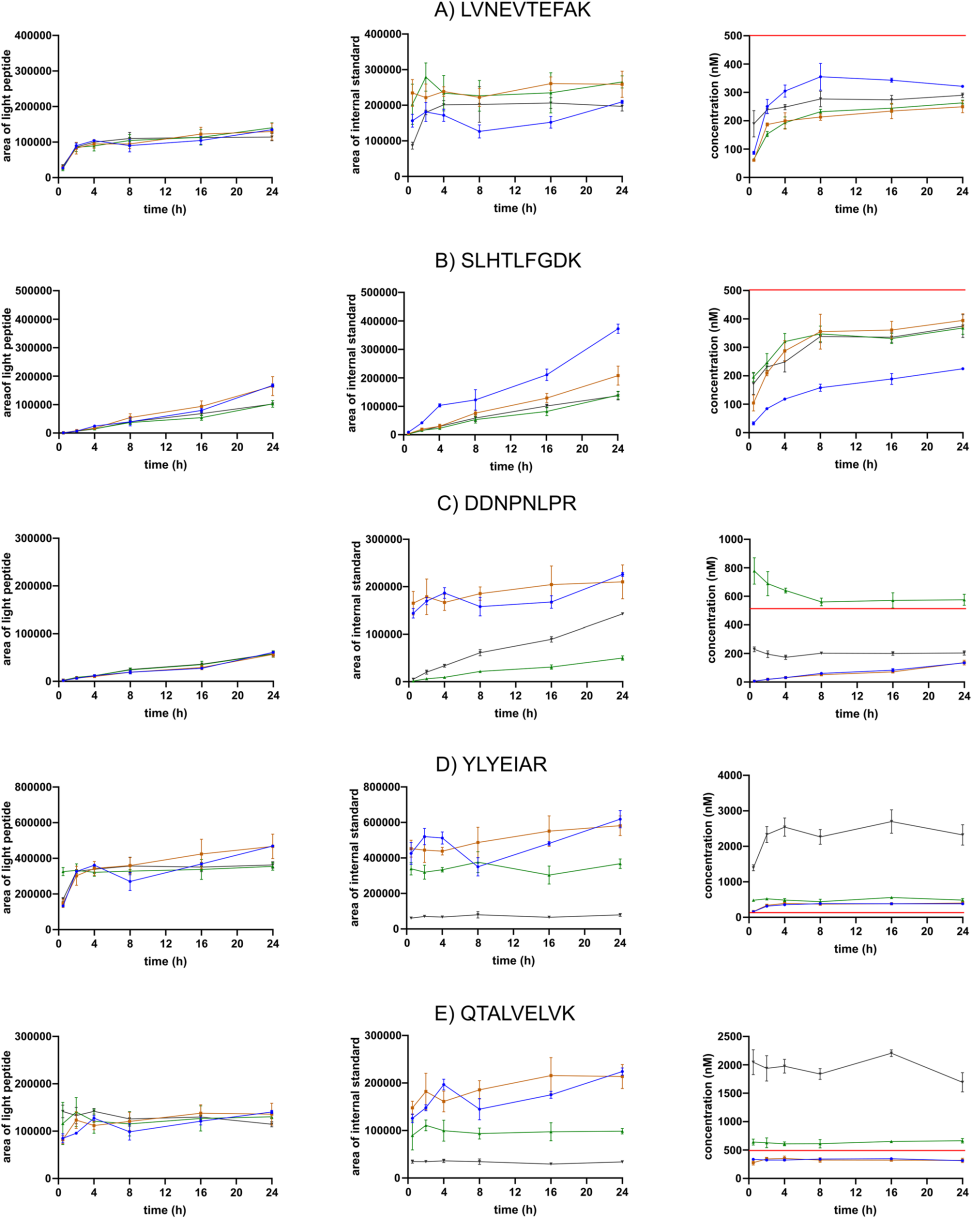
The trypsin digestion kinetics of signature peptides, LVNEVTEFAK (**A**), SLHTLFGDK (**B**), DDNPNLPR (**C**), YLYEIAR (**D**), and QTALVELVK (**E**). SIL-Ex peptide versions are color-coded: SIL-TCT (blue), SIL-ExC5 (orange), SIL-ExC3N3 (green), and SIL-ExC5N5 (black). For each signature peptide, the signal of light serum peptide (left panel) and SIL-Ex peptide (middle panel) is shown together with determined HSA concentration over digestion time (right panel). HSA concentration determined by reference methods (500 nM) indicated as a red line.

The enzymatic digestion efficiency severely affects the quantitative performance of a protein assay^10,11^. Previous studies demonstrated variable tryptic yields of peptides released from a protein. Overnight trypsin digestion is routinely used for reproducible proteolytic yields as the peptide's stability and formation efficiency may vary^11^. Maximal trypsinization yields of some signature peptides are reached within two hours^10,16^, while other peptides require longer digestion time^30^. We optimized the trypsin digestion duration to ensure reproducible signature peptide yields needed for a quantitative protein assay. We studied the signature peptide formation from light HSA protein and each version of SIL-Ex peptides after 0.5, 2, 4, 8, 16, and 24 h of incubation. SRM peak areas of light and heavy signature peptides and determined HSA protein concentrations (nM) were plotted against incubation time (h), comparing the efficiency of light HSA proteolysis with SIL-Ex peptides (Figs. 4, S3 and S4). The formation of HSA signature peptides LVNE, SLHT, DDNP, YLYE, and QTAL was compared for SIL-TCT, SIL-ExC5, SIL-ExC3N3, and SIL-ExC5N5 peptides (Fig. 4). Besides, the formation of HSA signature peptides DLGE, LCTV, LVTD, TYET, and FQNA was compared for SIL-TCT and SIL-ExC5 peptides only (Fig. S4).

Optimal surrogate peptides for quantification are formed quickly within the initial hours of enzymatic digestion and later produce a constant SRM response. In this study, five peptides (i.e., YLYE, LVNE, QTAL, LVTD, TYET) indicated fast kinetics reaching maximal signature peptide yields from light HSA within 2–4 h of incubation (Figs. 4, S3 and S4). SIL peptides indicated a similar trend of rapidly reaching plateau concentration. However, the signal of heavy signature peptides varied markedly depending on the type of SIL-Ex peptide (Figs. 4 and S4). Signature peptides TYET, LVTD, and LVNE, were formed from SIL-Ex peptides faster than from HSA protein (Fig. S4). The result was falsely low HSA concentration determined from the light-to-heavy signature peptide ratio. Digestion yields of signature peptides QTAL and YLYE formed from SIL-ExC5N5 peptides were lower, probably due to insufficient solubility in AmBic/SDC buffer with 5% ACN. On the other hand, SIL-ExC3N3 peptides and HSA protein's digestion efficiency was identical, except for signature peptide LVNE (Fig. 4).

The rate of signature peptide formation from target proteins has to be equal with SIL-Ex peptide for accurate quantification. Signature peptides SLHT, DLGE, DDNP, LCTV, and FQNA, exhibited slow formation from HSA protein (Figs. 4, S3 and S4). The digestion efficiencies of C-terminal extended SIL-TCT and SIL-ExC5 peptides DLGE, LCTV, and FQNA were much faster, reaching a plateau within the initial two hours (LCTV and DLGE) or FQNA even in 30 min (Fig. S4). We attribute the discordant peptide yields between HSA protein and SIL-Ex peptides to N-terminal extension, perhaps limiting the enzymatic digestion rate. Signature peptide DDNP available as C-terminal extended (i.e., SIL-TCT and SIL-ExC5) and both C- and N-termini extended (i.e., SIL-ExC3N3 and SIL-ExC5N5) supports the assumption. SIL peptides with only C-terminal extension rapidly formed signature peptide DDNP, in contrast to SIL peptides, elongated at both C- and N-termini, mimicking the slow digestion kinetics of light HSA protein (Fig. 4). DDNP and DLGE signature peptides reportedly do not reach a signal plateau even after 48 h^10^ as acidic amino acid residues near the cleavage site reduce trypsin efficiency^31–33^. Signature peptides containing a sequence motif causing slow trypsinization are not considered quantitative. However, we demonstrated an accurate quantification with slow-forming signature peptides using an adequately designed SIL-Ex peptide.

### Protein assays using extended SIL peptides and a recombinant SIL-HSA protein

We assessed the quantitative performance and reproducibility in optimal reconstitution buffers. We previously selected AmBic/SDC with 5% ACN for SIL-TCT, SIL-ExC5, and SIL-ExC3N3 peptides and AmBic/SDC with 50% ACN for hydrophobic SIL-ExC5N5 peptides. The HSA concentration determined using each type of SIL-Ex peptide was compared to the reference HSA concentration, the average of five signature peptides of SIL-HSA protein (445.9 nM). The HSA concentration determined using SIL peptides elongated only at C-terminus was highly inaccurate, as discussed earlier. SIL peptides elongated with (i) commercial trypsin cleavable tetrapeptide SAnYG tag and (ii) five amino acids of HSA protein’s natural sequence at C-terminus determined an average concentration of HSA at 268.6 nM and 274.3 nM, respectively (Table 1). SIL peptides extended only at C-terminus were demonstrated not suitable for absolute quantification.

**Table 1:** The comparison of the quantitative performance of SIL-HSA and various types of SIL-Ex peptides (SIL-Ex were reconstituted in AmBic/SDC buffer with 5% ACN except for SIL-ExC5N5 indicating optimal quantitative performance in AmBic/SDC buffer with 50% ACN).

Peptide	SIL-TCT		SIL-ExC5		SIL-ExC3N3		SIL-ExC5N5		SIL-HSA	
	nM	CV (%)	nM	CV (%)	nM	CV (%)	nM	CV (%)	nM	CV (%)
DDNPNLPR	82.50	14.05	71.61	3.41	589.77	7.39	223.41	9.07	431.64	8.33
SLHTLFGDK	188.88	10.21	361.20	8.52	400.10	11.23	347.96	0.95	473.12	5.10
YLYEIAR	382.39	2.33	383.26	3.87	567.83	14.64	523.83	2.99	468.11	9.15
LVNEVTEFAK	343.12	2.07	234.24	11.66	267.93	4.66	197.66	5.10	398.73	10.53
QTALVELVK	345.95	5.45	320.92	4.59	453.52	8.01	413.01	8.13	457.73	6.81
Average	268.57	6.82	274.25	6.41	432.18	9.19	341.17	5.25	445.86	7.98

**Figure 5:**
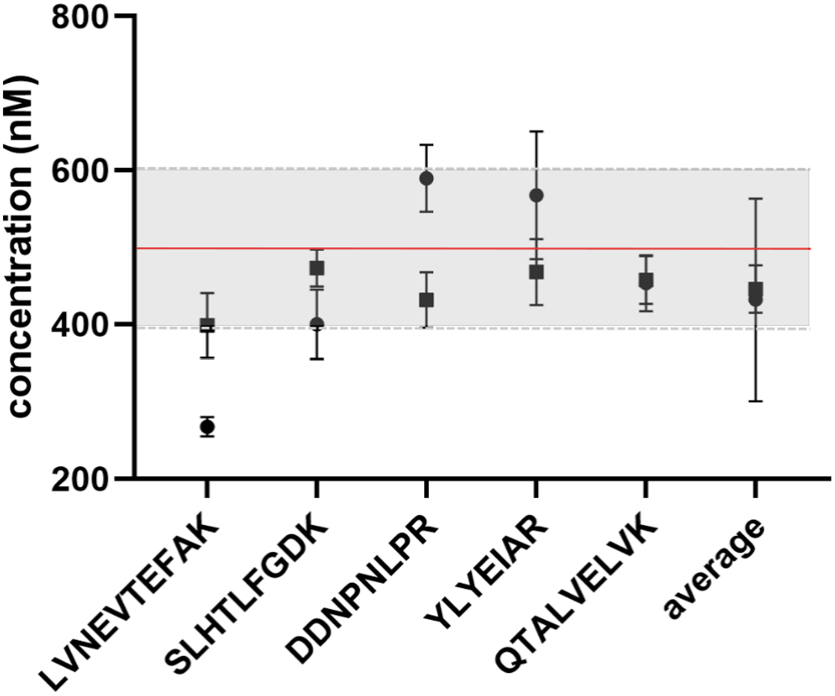
The comparison of the quantitative performance of SIL-ExC3N3 (filled circle) and SIL-HSA (filled square). HSA concentration determined by reference methods (500 nM) indicated as a red line; the grey area represents 20% tolerance.

The quantitative accuracy improved with the use of SIL peptides extended at both C- and N-termini (i.e., SIL-ExC3N3 and SIL-ExC5N5). The HSA concentration determined as the average of five SIL-ExC5N5 signature peptides was 341.2 nM and ranged from 197.7 to 523.8 nM for individual peptides (Table 1). SIL-ExC3N3 peptides determined average HSA concentration at 432.2 nM, nearly identical to SIL-HSA protein (445.9 nM, Table 1, and Fig. 5). Signature peptides SLHT, DDNP, YLYE, and QTAL, formed from SIL-HSA protein and SIL-ExC3N3 peptides accurately quantified HSA concentration (500 nM) within 20% tolerance from the reference BCG assay. Except for LVNE peptide determining the HSA protein concentration at 398.7 nM and 267.93 nM by SIL-HSA protein internal standard and SIL-ExC3N3 peptide, respectively (Fig. 5).

Previous comparative studies demonstrated superior quantitative performance of SIL proteins over tryptic SIL or SIL-Ex peptides^11–13,34^. However, the position (only C-terminus or both C- and N-termini) and the length of the extension varied among studies. For instance, Jiang et al. compared SIL peptides extended with three amino acids at C-terminus with SIL peptides extended with 2–5 amino acids at both C- and N-termini to report no significant differences in the quantitative performance^18^. However, the study was limited to comparing various types of SIL-Ex peptides with different signature peptides^18^. A study by Scott et al. investigated three types of amino acid extensions at both termini (extended with two, four, and six amino acids) in comparison to tryptic SIL peptides without extension^11^. The superior quantitative performance was attributed to SIL-Ex elongated with six amino acids. However, only two surrogate peptides were investigated. Our study confirmed that SIL proteins are indeed an optimal internal standard for absolute quantification. More importantly, we demonstrated that the SIL-ExC3N3 peptides are an alternative with identical quantitative performance as the SIL protein.

## Conclusions

This study is the first to evaluate the influence of the sequence extension's length and position on synthetic extended "winged" peptides’ quantitative performance. The HSA protein model was used with a representative, meticulously selected set of signature peptides to deliver robust and reliable results. We demonstrated the influence of the sequence extension on enzymatic digestion yields, solubility, and overall quantitative performance. The optimal quantitative performance of SIL-ExC3N3 peptides was verified with the independent reference methods (HSA protein concentration determined on SIL-HSA internal standard and BCG assay). We recommend using SIL-ExC3N3 peptides as an equally accurate but more available internal standard to SIL-proteins in quantitative proteomics.

## Supplementary Information

Supplementary Information.

## Data Availability

The mass spectrometry data were deposited to the PANORAMA Repository (https://panoramaweb.org/U%20of%20Masaryk%20-%20RECETOX/Stuchlikova_winged_raw/project-begin.view?pageId=Raw%20Data).
