# Supplementary material for: A comparative study of synthetic winged peptides for absolute protein quantification
Source: Sci Rep. 2021 May 25;11:10880. doi: 10.1038/s41598-021-90087-9 (PMC8149832; doi:10.1038/s41598-021-90087-9)
Supplement: Supplementary file 1 — Supplementary Information. [file 41598_2021_90087_MOESM1_ESM.docx]

Supplementary material for the manuscript:

A comparative study of synthetic winged peptides for absolute protein quantification

Eliska Benesova, Veronika Vidova, Zdenek Spacil*

Masaryk University, Faculty of Science, RECETOX, Kamenice 753/5, pavilion D29, 625 00 Brno, Czech Republic

Address correspondence to Dr. Zdenek Spacil, Masaryk University, Faculty of Science, Kamenice 753/5, pavilion D29, 625 00 Brno, Czech Republic. Tel. (+420) 549 49 7989; e-mail: [spacil@recetox.muni.cz](mailto:spacil@recetox.muni.cz) or spacil@u.washington.edu

**Table of contents**

**Supplementary results and discussion** - SPE recovery

**Supplementary results and discussion** - Reproducibility

**Figure S1.** Chromatograms of all candidate peptide surrogates with the position in the protein sequence indicated in brackets.

**Figure S2.** The trypsin digestion efficiency of all types of SIL extended peptides reconstituted in different solvents.

**Figure S3.** The best performing signature peptides for HSA protein in a serum sample plotted over incubation time with trypsin.

**Figure S4**. Trypsin digestion efficiency of signature peptides, DLGEENFK (A), LCTVATLR (B), LVTDLTK (C), TYETTLEK (D), and FQNALLVR (E).

**Figure S5.** Intra- and inter-day precision for all SIL extended peptides.

**Table S1.** Sequences of selected SIL extended peptides, average molecular weights, and quantifier transitions.

**Table S2.** Dynamic SRM library with experimental retention times and collision energies.

**Table S3.** Library of initially screened transitions generated in SRMAtlas.

**Table S4.** Library of initially screened transitions generated in Skyline software.

**Table S5.** List of all candidate peptides selected with the guidance of SRM Atlas (13 peptides) and/or Skyline software (24 peptides) with the position of the peptide in protein sequence, precursor and product ions, experimental retention times, and peak areas in the initial screening in a 100-fold diluted human serum sample.

**Table S6.** Hydrophobicity indexes and predicted water solubility of signature and SIL extended peptides ordered by increasing hydrophobicity.

**Table S7.** SIL-TCT and SIL-ExC5 peptides' quantitative performance reconstituted in water with 5% ACN and AmBic/SDC buffer with 5% ACN.

**Table S8.** SPE recoveries of light serum peptides, SIL-HSA, and heavy/light peptide ratios.

**Supplementary results and discussion**

**SPE recovery.** To evaluate SPE recovery, samples (n = 3) processed with SPE were compared to samples (n = 3) not subjected to SPE desalting. SIL-HSA was used as an internal standard. SPE recovery was calculated as a ratio between SRM peak areas of a peptide in the sample with SPE to the sample without SPE. Recoveries were calculated for both light serum HSA and SIL-HSA. The average SPE recovery for light HSA was 72.35%, with the lowest recovery for peptide FQNA (62.91%) and highest for peptide DDNP (81.34%, Table S8). Similar trends were observed for the SIL-HSA with an average recovery of 69.88%, lowest for FQNA (60.19%), and highest for DDNP (76.54%, Table S8). SPE recovery for light HSA and SIL-HSA was identical for all peptides, and the average ratio between SIL-HSA recovery and light HSA recovery was 0.97. All recoveries for light HSA peptides, SIL-HSA peptides, and ratios are shown in Table S8.

**Reproducibility.** The intra-day (n = 3) and inter-day (n = 9) assay precision was performed separately for each type of SIL extended peptides. The pooled serum sample was spiked with a mixture of SIL extended peptides, processed as previously described, and analyzed using UHPLC-SRM assay at three different time points during five months. Average HSA concentrations determined using various SIL extended peptides, CVs (%) for each measurement, and the inter-day precision are shown in Fig. S5. In general, the average CV for intraday precision (n = 3) was between 5.34% and 8.63%, showing a very similar trend for all investigated SIL peptides. For the interday precision, CV was between 9.74% and 17.17% (Fig. S5).

**Fig S1.** Chromatograms of all candidate peptide surrogates with the position in the protein sequence indicated in brackets. Sequences of peptides selected for further investigation are marked in bold. Four peptides selected under Skyline software guidance (CCAAADPHECYAK, VHTECCHGDLLECADDR, RPCFSALEVDETYVPK, ETCFAEEGK) were not detected in the initial UHPLC-MS screening.


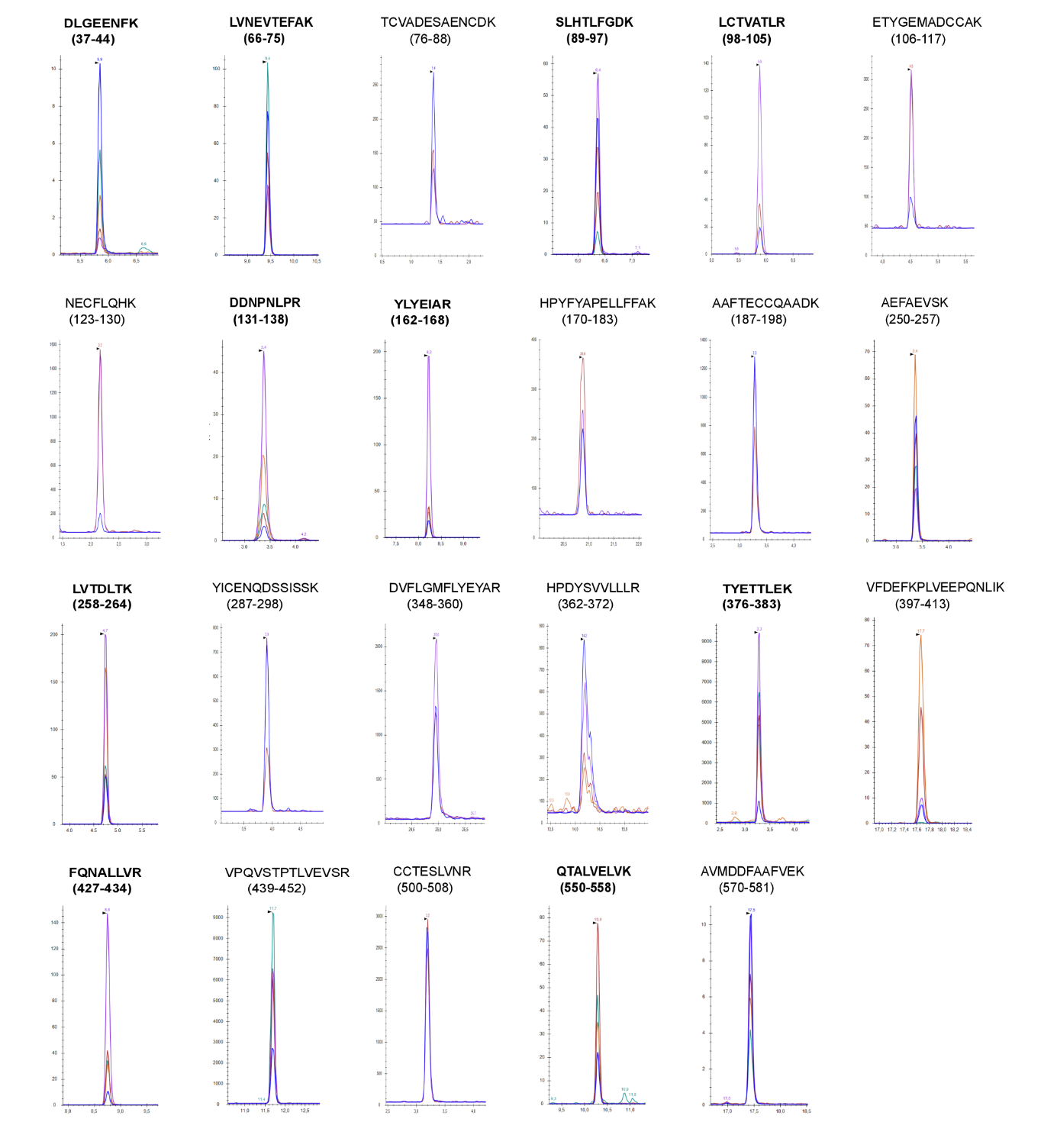


**Fig. S2.** The trypsin digestion efficiency of all SIL extended peptides reconstituted in various solvents. The peak area of light serum peptide with spiked SIL-Ex peptide reconstituted in AmBic/SDC buffer with 5% ACN was used as the reference value (100%). The peptide sequence hydrophobicity increases in the directions of red arrows.


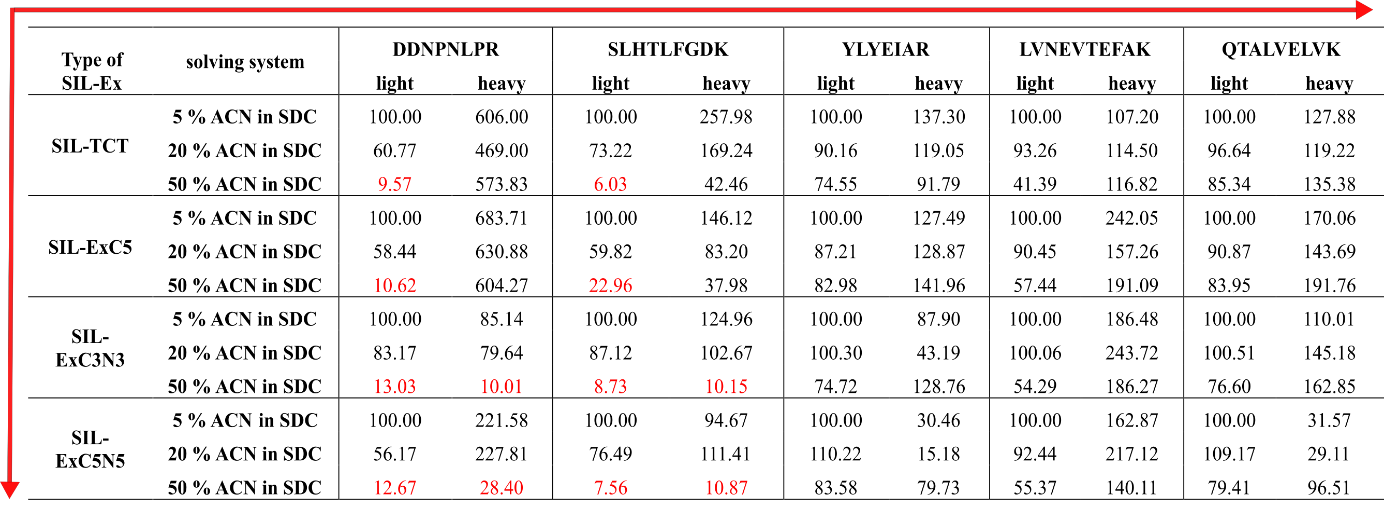


**Fig. S3.** The best performing signature peptides for HSA protein in a serum sample plotted over incubation time with trypsin. Types of SIL extended peptides are color-coded: SIL-TCT (blue), SIL- ExC5 (orange), SIL-ExC3N3 (green), and SIL-ExC5N5 (black).


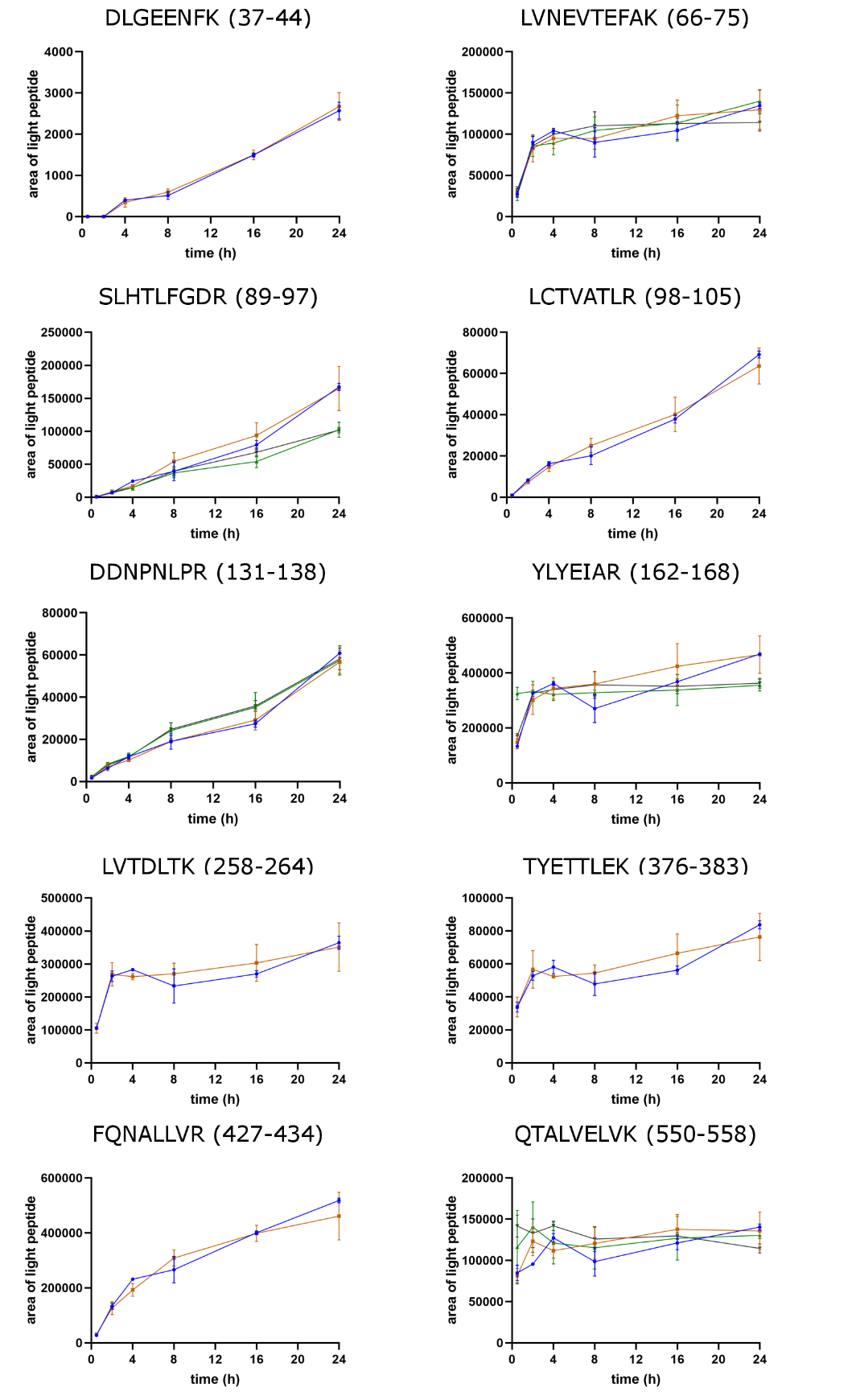


**Fig. S4**. Trypsin digestion efficiency of signature peptides, DLGEENFK (A), LCTVATLR (B), LVTDLTK (C), TYETTLEK (D), and FQNALLVR (E). SIL extended peptides are color-coded: SIL-TCT (blue) and SIL- ExC5 (orange). For each peptide, the signal of light serum peptide (left panel) and SIL-Ex peptide (middle panel) is shown together with determined HSA concentration over digestion time (right panel). HSA concentration determined by reference methods (500 nM) indicated as a red line.


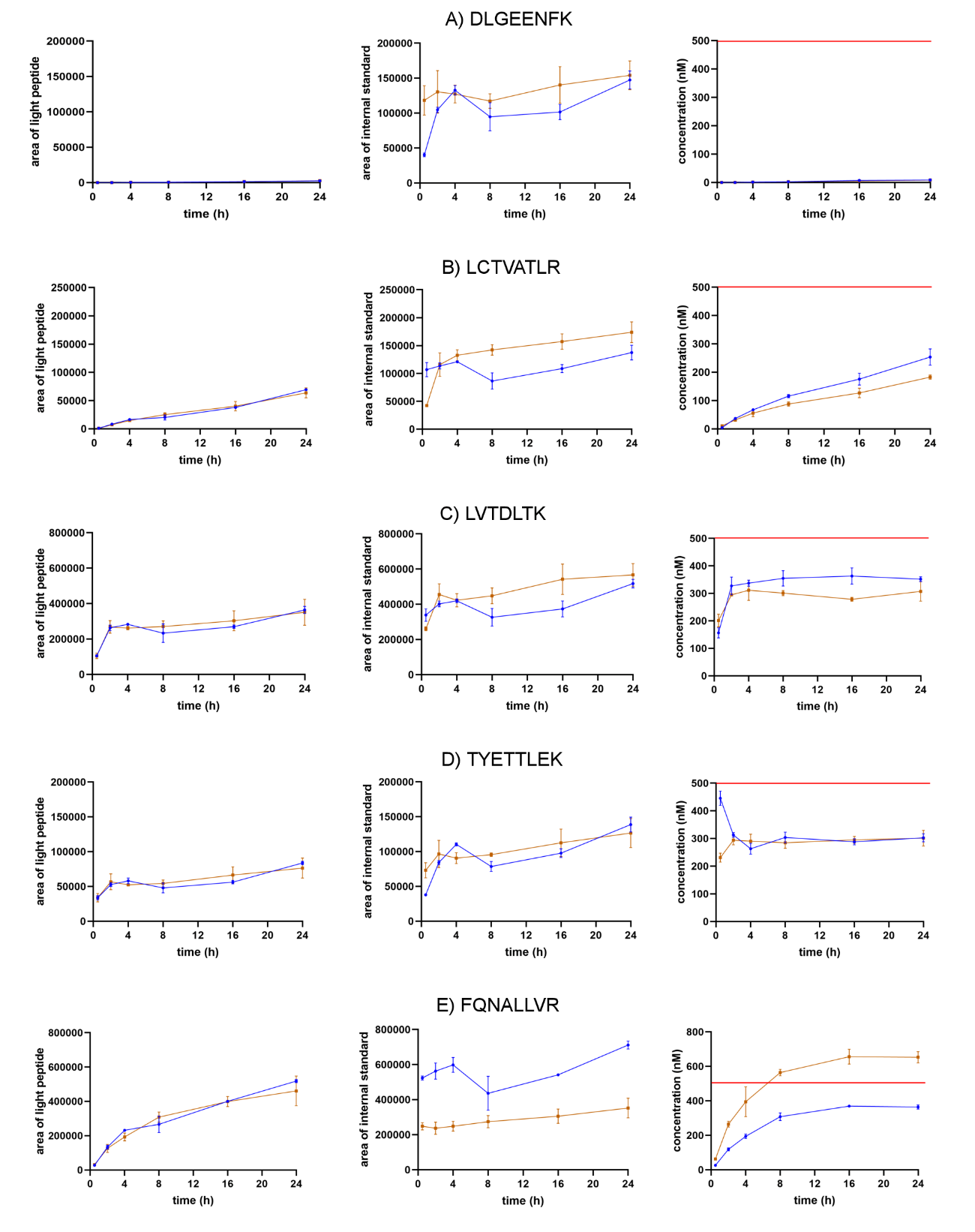


**Fig. S5.** Intra- and inter-day precision for all types of SIL extended peptides.





**Table S1.** Sequences of selected SIL extended peptides, average molecular weight, and quantifier transitions. For SIL-TCT peptides, average molecular weight includes a commercial Qtag (Mw 423.38; sequence: SAnYG; nY = nitrotyrosine). Isotopically labeled lysine and arginine in the sequence are marked in bold and alkylated cysteines are marked with asterisk.

| **Type of SIL-Ex** | **SIL-Ex sequence** | **Average Mw** | **Quantifier transition** |
| --- | --- | --- | --- |
| SIL-TCT | DLGEENF**K**SAnYG | 1,382.30 | 480.23 -> 731.35 |
|  | LVNEVTEFA**K**SAnYG | 1,580.60 | 579.32 -> 945.48 |
|  | SLHTLFGD**K**SAnYG | 1,448.46 | 342.52 -> 327.18 |
|  | LC*TVATL**R**SAnYG | 1,366.43 | 472.27 -> 670.41 |
|  | DDNPNLP**R**SAnYG | 1,373.28 | 475.73 -> 606.36 |
|  | YLYEIA**R**SAnYG | 1,360.37 | 469.25 -> 661.35 |
|  | LVTDLT**K**SAnYG | 1,220.24 | 399.25 -> 585.33 |
|  | TYETTLE**K**SAnYG | 1,415.36 | 496.75 -> 728.39 |
|  | FQNALLV**R**SAnYG | 1,393.44 | 485.79 -> 695.44 |
|  | QTALVELV**K**SAnYG | 1,431.50 | 504.81 -> 595.39 |
| SIL-ExC5 | DLGEENF**K**ALVLI | 1,468.62 | 480.23 -> 731.35 |
|  | LVNEVTEFA**K**TC*VAD | 1,703.83 | 579.32 -> 945.48 |
|  | SLHTLFGD**K**LC*TVA | 1,569.76 | 342.52 -> 327.18 |
|  | LC*TVATL**R**ETYGE | 1,522.61 | 472.27 -> 670.41 |
|  | DDNPNLP**R**LVRPE | 1,544.62 | 475.73 -> 606.36 |
|  | YLYEIA**R**RHPYF | 1,637.81 | 469.25 -> 661.35 |
|  | LVTDLT**K**VHTEC* | 1,423.55 | 399.25 -> 585.33 |
|  | TYETTLE**K**C*C*AAA | 1,525.63 | 496.75 -> 728.39 |
|  | FQNALLV**R**YTKKV | 1,589.82 | 485.79 -> 695.44 |
|  | QTALVELV**K**HKPKA | 1,569.81 | 504.81 -> 595.39 |
| SIL-ExC3N3 | HVKLVNEVTEFA**K**TC*V | 1,882.10 | 579.32 -> 945.48 |
|  | C*DKSLHTLFGD**K**LC*T | 1,803.01 | 342.52 -> 327.18 |
|  | LQHKDDNPNLP**R**LVR | 1,824.99 | 475.73 -> 606.36 |
|  | LKKYLYEIA**R**RHP | 1,696.95 | 469.25 -> 661.35 |
|  | IKKQTALVELV**K**HKP | 1,740.06 | 504.81 -> 595.39 |
| SIL-ExC5N5 | FEDHVKLVNEVTEFA**K**TC*VAD | 2,459.65 | 579.32 -> 945.48 |
|  | AENC*DKSLHTLFGD**K**LC*TVA | 2,287.51 | 342.52 -> 327.18 |
|  | FLQHKDDNPNLP**R**LVRPE | 2,198.40 | 475.73 -> 606.36 |
|  | TFLKKYLYEIA**R**RHPYF | 2,255.59 | 469.25 -> 661.35 |
|  | RQIKKQTALVELV**K**HKPKA | 2,223.63 | 504.81 -> 595.39 |

**Table S2.** Dynamic SRM library with experimental retention times and collision energies.

| **Peptide name** | **Ion name** | **Precursor Ion** | **Product Ion** | **Retention Time (min)** | **Collision Energy** |
| --- | --- | --- | --- | --- | --- |
| DDNPNLPR light | b2 | 470.73 | 231.06 | 3.4 | 20.5 |
| DDNPNLPR light | y2 | 470.73 | 272.17 | 3.4 | 20.5 |
| DDNPNLPR light | y4 | 470.73 | 499.30 | 3.4 | 20.5 |
| DDNPNLPR light | y5 | 470.73 | 596.35 | 3.4 | 20.5 |
| DDNPNLPR light | y6 | 470.73 | 710.39 | 3.4 | 20.5 |
| DDNPNLPR heavy | b2 | 475.73 | 231.06 | 3.4 | 21 |
| DDNPNLPR heavy | y2 | 475.73 | 282.18 | 3.4 | 21 |
| DDNPNLPR heavy | y4 | 475.73 | 509.31 | 3.4 | 21 |
| DDNPNLPR heavy | y5 | 475.73 | 606.36 | 3.4 | 21 |
| DDNPNLPR heavy | y6 | 475.73 | 720.40 | 3.4 | 21 |
| DLGEENFK light | b2 | 476.23 | 229.12 | 5.9 | 20.7 |
| DLGEENFK light | y2 | 476.23 | 294.18 | 5.9 | 20.7 |
| DLGEENFK light | y4 | 476.23 | 537.27 | 5.9 | 20.7 |
| DLGEENFK light | y5 | 476.23 | 666.31 | 5.9 | 20.7 |
| DLGEENFK light | y6 | 476.23 | 723.33 | 5.9 | 20.7 |
| DLGEENFK heavy | b2 | 480.23 | 229.12 | 5.9 | 21 |
| DLGEENFK heavy | y2 | 480.23 | 302.19 | 5.9 | 21 |
| DLGEENFK heavy | y4 | 480.23 | 545.28 | 5.9 | 21 |
| DLGEENFK heavy | y5 | 480.23 | 674.32 | 5.9 | 21 |
| DLGEENFK heavy | y6 | 480.23 | 731.34 | 5.9 | 21 |
| FQNALLVR light | y3 | 480.79 | 387.27 | 8.8 | 20.8 |
| FQNALLVR light | y4 | 480.79 | 500.36 | 8.8 | 20.8 |
| FQNALLVR light | y5 | 480.79 | 571.39 | 8.8 | 20.8 |
| FQNALLVR light | y6 | 480.79 | 685.44 | 8.8 | 20.8 |
| FQNALLVR light | y7 | 480.79 | 813.49 | 8.8 | 20.8 |
| FQNALLVR heavy | y3 | 485.79 | 397.28 | 8.8 | 21 |
| FQNALLVR heavy | y4 | 485.79 | 510.37 | 8.8 | 21 |
| FQNALLVR heavy | y5 | 485.79 | 581.40 | 8.8 | 21 |
| FQNALLVR heavy | y6 | 485.79 | 695.45 | 8.8 | 21 |
| FQNALLVR heavy | y7 | 485.79 | 823.50 | 8.8 | 21 |
| LCTVATLR light | y5 | 467.26 | 559.36 | 5.9 | 27.8 |
| LCTVATLR light | y6 | 467.26 | 660.40 | 5.9 | 27.8 |
| LCTVATLR light | y7 | 467.26 | 820.43 | 5.9 | 27.8 |
| LCTVATLR heavy | y5 | 472.27 | 569.36 | 5.9 | 28 |
| LCTVATLR heavy | y6 | 472.27 | 670.41 | 5.9 | 28 |
| LCTVATLR heavy | y7 | 472.27 | 830.44 | 5.9 | 28 |
| LVNEVTEFAK light | b2 | 575.31 | 213.16 | 9.4 | 24 |
| LVNEVTEFAK light | y3 | 575.31 | 365.22 | 9.4 | 24 |
| LVNEVTEFAK light | y5 | 575.31 | 595.31 | 9.4 | 24 |
| LVNEVTEFAK light | y6 | 575.31 | 694.38 | 9.4 | 24 |
| LVNEVTEFAK light | y8 | 575.31 | 937.46 | 9.4 | 24 |
| LVNEVTEFAK heavy | b2 | 579.32 | 213.16 | 9.4 | 24 |
| LVNEVTEFAK heavy | y3 | 579.32 | 373.23 | 9.4 | 24 |
| LVNEVTEFAK heavy | y5 | 579.32 | 603.32 | 9.4 | 24 |
| LVNEVTEFAK heavy | y6 | 579.32 | 702.39 | 9.4 | 24 |
| LVNEVTEFAK heavy | y8 | 579.32 | 945.48 | 9.4 | 24 |
| LVTDLTK light | b2 | 395.24 | 213.16 | 4.7 | 18 |
| LVTDLTK light | y2 | 395.24 | 248.16 | 4.7 | 18 |
| LVTDLTK light | y3 | 395.24 | 361.25 | 4.7 | 18 |
| LVTDLTK light | y5 | 395.24 | 577.32 | 4.7 | 18 |
| LVTDLTK light | y6 | 395.24 | 676.39 | 4.7 | 18 |
| LVTDLTK heavy | b2 | 399.25 | 213.16 | 4.7 | 18 |
| LVTDLTK heavy | y2 | 399.25 | 256.17 | 4.7 | 18 |
| LVTDLTK heavy | y3 | 399.25 | 369.26 | 4.7 | 18 |
| LVTDLTK heavy | y5 | 399.25 | 585.33 | 4.7 | 18 |
| LVTDLTK heavy | y6 | 399.25 | 684.40 | 4.7 | 18 |
| QTALVELVK light | y2 | 500.81 | 246.18 | 10.3 | 21.4 |
| QTALVELVK light | y4 | 500.81 | 488.31 | 10.3 | 21.4 |
| QTALVELVK light | y5 | 500.81 | 587.38 | 10.3 | 21.4 |
| QTALVELVK light | y6 | 500.81 | 700.46 | 10.3 | 21.4 |
| QTALVELVK light | y7 | 500.81 | 771.5 | 10.3 | 21.4 |
| QTALVELVK heavy | y2 | 504.82 | 254.19 | 10.3 | 22 |
| QTALVELVK heavy | y4 | 504.82 | 496.32 | 10.3 | 22 |
| QTALVELVK heavy | y5 | 504.82 | 595.39 | 10.3 | 22 |
| QTALVELVK heavy | y6 | 504.82 | 708.47 | 10.3 | 22 |
| QTALVELVK heavy | y7 | 504.82 | 779.51 | 10.3 | 22 |
| SLHTLFGDK light | b4 | 339.85 | 439.23 | 6.4 | 7.4 |
| SLHTLFGDK light | y3 | 339.85 | 319.16 | 6.4 | 7.4 |
| SLHTLFGDK light | y4 | 339.85 | 233.62 | 6.4 | 7.4 |
| SLHTLFGDK light | y4 | 339.85 | 466.23 | 6.4 | 7.4 |
| SLHTLFGDK light | y7 | 339.85 | 409.21 | 6.4 | 7.4 |
| SLHTLFGDK heavy | b4 | 342.52 | 439.23 | 6.4 | 7.4 |
| SLHTLFGDK heavy | y3 | 342.52 | 327.18 | 6.4 | 7.4 |
| SLHTLFGDK heavy | y4 | 342.52 | 237.63 | 6.4 | 7.4 |
| SLHTLFGDK heavy | y4 | 342.52 | 474.24 | 6.4 | 7.4 |
| SLHTLFGDK heavy | y7 | 342.52 | 413.22 | 6.4 | 7.4 |
| TYETTLEK light | b2 | 492.75 | 265.12 | 3.3 | 21.2 |
| TYETTLEK light | y2 | 492.75 | 276.16 | 3.3 | 21.2 |
| TYETTLEK light | y5 | 492.75 | 591.34 | 3.3 | 21.2 |
| TYETTLEK light | y6 | 492.75 | 720.38 | 3.3 | 21.2 |
| TYETTLEK light | y7 | 492.75 | 883.44 | 3.3 | 21.2 |
| TYETTLEK heavy | b2 | 496.76 | 265.12 | 3.3 | 21 |
| TYETTLEK heavy | y2 | 496.76 | 284.17 | 3.3 | 21 |
| TYETTLEK heavy | y5 | 496.76 | 599.35 | 3.3 | 21 |
| TYETTLEK heavy | y6 | 496.76 | 728.39 | 3.3 | 21 |
| TYETTLEK heavy | y7 | 496.76 | 891.45 | 3.3 | 21 |
| YLYEIAR light | b2 | 464.25 | 277.16 | 8.2 | 20.3 |
| YLYEIAR light | y3 | 464.25 | 359.24 | 8.2 | 20.3 |
| YLYEIAR light | y4 | 464.25 | 488.28 | 8.2 | 20.3 |
| YLYEIAR light | y5 | 464.25 | 651.35 | 8.2 | 20.3 |
| YLYEIAR light | y6 | 464.25 | 764.43 | 8.2 | 20.3 |
| YLYEIAR heavy | b2 | 469.25 | 277.16 | 8.2 | 21 |
| YLYEIAR heavy | y3 | 469.25 | 369.25 | 8.2 | 21 |
| YLYEIAR heavy | y4 | 469.25 | 498.29 | 8.2 | 21 |
| YLYEIAR heavy | y5 | 469.25 | 661.36 | 8.2 | 21 |
| YLYEIAR heavy | y6 | 469.25 | 774.44 | 8.2 | 21 |

**Table S3.** Library of initially screened transitions generated in SRMAtlas.

| **Peptide name** | **Ion name** | **Precursor Ion** | **Product Ion** | **Collision Energy** |
| --- | --- | --- | --- | --- |
| AEFAEVSK | b2 | 440.72 | 201.09 | 19.6 |
| AEFAEVSK | b3 | 440.72 | 348.16 | 19.6 |
| AEFAEVSK | y2 | 440.72 | 234.15 | 19.6 |
| AEFAEVSK | y5 | 440.72 | 533.29 | 19.6 |
| AEFAEVSK | y6 | 440.72 | 680.36 | 19.6 |
| AVMDDFAAFVEK | y10 | 671.82 | 1172.53 | 26.4 |
| AVMDDFAAFVEK | y4 | 671.82 | 522.29 | 26.4 |
| AVMDDFAAFVEK | y5 | 671.82 | 593.33 | 26.4 |
| AVMDDFAAFVEK | y6 | 671.82 | 664.37 | 26.4 |
| AVMDDFAAFVEK | y9 | 671.82 | 1041.49 | 26.4 |
| DDNPNLPR | b2 | 470.73 | 231.06 | 20.5 |
| DDNPNLPR | y2 | 470.73 | 272.17 | 20.5 |
| DDNPNLPR | y4 | 470.73 | 499.30 | 20.5 |
| DDNPNLPR | y5 | 470.73 | 596.35 | 20.5 |
| DDNPNLPR | y6 | 470.73 | 710.39 | 20.5 |
| DLGEENFK | b2 | 476.23 | 229.12 | 20.7 |
| DLGEENFK | y2 | 476.23 | 294.18 | 20.7 |
| DLGEENFK | y4 | 476.23 | 537.27 | 20.7 |
| DLGEENFK | y5 | 476.23 | 666.31 | 20.7 |
| DLGEENFK | y6 | 476.23 | 723.33 | 20.7 |
| FQNALLVR | y3 | 480.79 | 387.27 | 20.8 |
| FQNALLVR | y4 | 480.79 | 500.36 | 20.8 |
| FQNALLVR | y5 | 480.79 | 571.39 | 20.8 |
| FQNALLVR | y6 | 480.79 | 685.44 | 20.8 |
| FQNALLVR | y7 | 480.79 | 813.49 | 20.8 |
| LVNEVTEFAK | b2 | 575.31 | 213.16 | 23.6 |
| LVNEVTEFAK | y3 | 575.31 | 365.22 | 23.6 |
| LVNEVTEFAK | y5 | 575.31 | 595.31 | 23.6 |
| LVNEVTEFAK | y6 | 575.31 | 694.38 | 23.6 |
| LVNEVTEFAK | y8 | 575.31 | 937.46 | 23.6 |
| LVTDLTK | b2 | 395.24 | 213.16 | 18.3 |
| LVTDLTK | y2 | 395.24 | 248.16 | 18.3 |
| LVTDLTK | y3 | 395.24 | 361.25 | 18.3 |
| LVTDLTK | y5 | 395.24 | 577.32 | 18.3 |
| LVTDLTK | y6 | 395.24 | 676.39 | 18.3 |
| QTALVELVK | y2 | 500.81 | 246.18 | 21.4 |
| QTALVELVK | y4 | 500.81 | 488.31 | 21.4 |
| QTALVELVK | y5 | 500.81 | 587.38 | 21.4 |
| QTALVELVK | y6 | 500.81 | 700.46 | 21.4 |
| QTALVELVK | y7 | 500.81 | 771.50 | 21.4 |
| SLHTLFGDK | b4 | 339.85 | 439.23 | 7.4 |
| SLHTLFGDK | y3 | 339.85 | 319.16 | 7.4 |
| SLHTLFGDK | y4 | 339.85 | 466.23 | 7.4 |
| SLHTLFGDK | y4 | 339.85 | 233.62 | 7.4 |
| SLHTLFGDK | y7 | 339.85 | 409.21 | 7.4 |
| TYETTLEK | b2 | 492.75 | 265.12 | 21.2 |
| TYETTLEK | y2 | 492.75 | 276.16 | 21.2 |
| TYETTLEK | y5 | 492.75 | 591.34 | 21.2 |
| TYETTLEK | y6 | 492.75 | 720.38 | 21.2 |
| TYETTLEK | y7 | 492.75 | 883.44 | 21.2 |
| VFDEFKPLVEEPQNLIK | y11 | 682.37 | 1279.73 | 19.8 |
| VFDEFKPLVEEPQNLIK | y15 | 682.37 | 899.98 | 19.8 |
| VFDEFKPLVEEPQNLIK | y4 | 682.37 | 244.17 | 19.8 |
| VFDEFKPLVEEPQNLIK | y6 | 682.37 | 712.44 | 19.8 |
| VFDEFKPLVEEPQNLIK | y8 | 682.37 | 970.52 | 19.8 |
| VPQVSTPTLVEVSR | b3 | 756.43 | 325.19 | 28.9 |
| VPQVSTPTLVEVSR | y10 | 756.43 | 1088.60 | 28.9 |
| VPQVSTPTLVEVSR | y11 | 756.43 | 1187.66 | 28.9 |
| VPQVSTPTLVEVSR | y13 | 756.43 | 706.89 | 28.9 |
| VPQVSTPTLVEVSR | y8 | 756.43 | 900.52 | 28.9 |
| YLYEIAR | b2 | 464.25 | 277.16 | 20.3 |
| YLYEIAR | y3 | 464.25 | 359.24 | 20.3 |
| YLYEIAR | y4 | 464.25 | 488.28 | 20.3 |
| YLYEIAR | y5 | 464.25 | 651.35 | 20.3 |
| YLYEIAR | y6 | 464.25 | 764.43 | 20.3 |

**Table S4.** Library of initially screened transitions generated in Skyline software.

| **Peptide name** | **Ion name** | **Precursor Ion** | **Product Ion** | **Collision Energy** |
| --- | --- | --- | --- | --- |
| LVNEVTEFAK | y7 | 575.31 | 823.42 | 21.9 |
| LVNEVTEFAK | y6 | 575.31 | 694.38 | 21.9 |
| LVNEVTEFAK | y5 | 575.31 | 595.31 | 21.9 |
| TCVADESAENCDK | y9 | 749.79 | 1067.39 | 27.8 |
| TCVADESAENCDK | y8 | 749.79 | 952.37 | 27.8 |
| TCVADESAENCDK | y7 | 749.79 | 823.33 | 27.8 |
| SLHTLFGDK | y7 | 509.27 | 817.42 | 19.6 |
| SLHTLFGDK | y6 | 509.27 | 680.36 | 19.6 |
| SLHTLFGDK | y5 | 509.27 | 579.31 | 19.6 |
| LCTVATLR | y7 | 467.26 | 820.43 | 18.2 |
| LCTVATLR | y6 | 467.26 | 660.40 | 18.2 |
| LCTVATLR | y5 | 467.26 | 559.36 | 18.2 |
| ETYGEMADCCAK | y8 | 717.77 | 984.36 | 26.7 |
| ETYGEMADCCAK | y7 | 717.77 | 855.32 | 26.7 |
| ETYGEMADCCAK | y6 | 717.77 | 724.28 | 26.7 |
| NECFLQHK | y7 | 538.25 | 961.46 | 20.6 |
| NECFLQHK | y6 | 538.25 | 832.41 | 20.6 |
| NECFLQHK | y5 | 538.25 | 672.38 | 20.6 |
| DDNPNLPR | y6 | 470.73 | 710.39 | 18.3 |
| DDNPNLPR | y5 | 470.73 | 596.35 | 18.3 |
| DDNPNLPR | y4 | 470.73 | 499.30 | 18.3 |
| HPYFYAPELLFFAK | y10 | 871.95 | 1198.65 | 31.9 |
| HPYFYAPELLFFAK | y9 | 871.95 | 1035.59 | 31.9 |
| HPYFYAPELLFFAK | y8 | 871.95 | 964.55 | 31.9 |
| AAFTECCQAADK | y8 | 686.29 | 981.38 | 25.6 |
| AAFTECCQAADK | y7 | 686.29 | 852.33 | 25.6 |
| AAFTECCQAADK | y6 | 686.29 | 692.30 | 25.6 |
| AEFAEVSK | y6 | 440.72 | 680.36 | 17.3 |
| AEFAEVSK | y5 | 440.72 | 533.29 | 17.3 |
| AEFAEVSK | y4 | 440.72 | 462.26 | 17.3 |
| VHTECCHGDLLECADDR | y11 | 1043.92 | 1300.56 | 37.7 |
| VHTECCHGDLLECADDR | y10 | 1043.92 | 1163.50 | 37.7 |
| VHTECCHGDLLECADDR | y9 | 1043.92 | 1106.48 | 37.7 |
| YICENQDSISSK | y9 | 722.32 | 1007.46 | 26.8 |
| YICENQDSISSK | y8 | 722.32 | 878.42 | 26.8 |
| YICENQDSISSK | y7 | 722.32 | 764.38 | 26.8 |
| DVFLGMFLYEYAR | y8 | 812.40 | 1092.52 | 29.9 |
| DVFLGMFLYEYAR | y7 | 812.40 | 961.48 | 29.9 |
| DVFLGMFLYEYAR | y6 | 812.40 | 814.41 | 29.9 |
| HPDYSVVLLLR | y10 | 656.37 | 1174.68 | 24.6 |
| HPDYSVVLLLR | y8 | 656.37 | 962.60 | 24.6 |
| HPDYSVVLLLR | y7 | 656.37 | 799.54 | 24.6 |
| HPDYSVVLLLR | y6 | 656.37 | 712.51 | 24.6 |
| TYETTLEK | y7 | 492.75 | 883.44 | 19.1 |
| TYETTLEK | y6 | 492.75 | 720.38 | 19.1 |
| TYETTLEK | y5 | 492.75 | 591.33 | 19.1 |
| CCAAADPHECYAK | y8 | 776.80 | 1019.43 | 28.7 |
| CCAAADPHECYAK | y7 | 776.80 | 904.40 | 28.7 |
| CCAAADPHECYAK | y6 | 776.80 | 807.35 | 28.7 |
| VFDEFKPLVEEPQNLIK | y11 | 1023.05 | 1279.73 | 37 |
| VFDEFKPLVEEPQNLIK | y10 | 1023.05 | 1182.67 | 37 |
| VFDEFKPLVEEPQNLIK | y9 | 1023.05 | 1069.59 | 37 |
| VFDEFKPLVEEPQNLIK | y6 | 1023.05 | 712.44 | 37 |
| FQNALLVR | y6 | 480.78 | 685.44 | 18.7 |
| FQNALLVR | y5 | 480.78 | 571.39 | 18.7 |
| FQNALLVR | y4 | 480.78 | 500.36 | 18.7 |
| VPQVSTPTLVEVSR | y13 | 756.43 | 1412.77 | 28 |
| VPQVSTPTLVEVSR | y9 | 756.43 | 1001.56 | 28 |
| VPQVSTPTLVEVSR | y8 | 756.43 | 900.51 | 28 |
| VPQVSTPTLVEVSR | y7 | 756.43 | 803.46 | 28 |
| CCTESLVNR | y7 | 569.75 | 818.44 | 21.7 |
| CCTESLVNR | y6 | 569.75 | 717.39 | 21.7 |
| CCTESLVNR | y5 | 569.75 | 588.35 | 21.7 |
| RPCFSALEVDETYVPK | y11 | 955.97 | 1263.65 | 34.8 |
| RPCFSALEVDETYVPK | y10 | 955.97 | 1192.61 | 34.8 |
| RPCFSALEVDETYVPK | y9 | 955.97 | 1079.53 | 34.8 |
| QTALVELVK | y7 | 500.81 | 771.50 | 19.3 |
| QTALVELVK | y6 | 500.81 | 700.46 | 19.3 |
| QTALVELVK | y5 | 500.81 | 587.38 | 19.3 |
| AVMDDFAAFVEK | y9 | 671.82 | 1041.49 | 25.1 |
| AVMDDFAAFVEK | y8 | 671.82 | 926.46 | 25.1 |
| AVMDDFAAFVEK | y7 | 671.82 | 811.43 | 25.1 |
| ETCFAEEGK | y8 | 535.73 | 941.40 | 20.5 |
| ETCFAEEGK | y7 | 535.73 | 840.36 | 20.5 |
| ETCFAEEGK | y6 | 535.73 | 680.32 | 20.5 |

**Table S5.** List of all candidate peptides selected with the guidance of SRM Atlas (13 peptides) and/or Skyline software (24 peptides) with the position of the peptide in protein sequence, precursor and product ions, experimental retention times, and peak areas in the initial screening in a 100-fold diluted human serum sample. Peptides selected for further investigation (10) are marked in bold.

| **Used software** | **Surrogate peptide sequence** | **Surrogate peptide position in HSA protein** | **SRM precursor ion [m/z]** | **SRM product ion [m/z]** | **Experimental retention time** | **Integrated peak area** |  |
| --- | --- | --- | --- | --- | --- | --- | --- |
|  |  |  |  |  |  |  |  |
| SRM atlas | **DLGEENFK** | **37-44** | **476.22++** | **723.33+** | **5.9** | **46,222** |  |
|  | **LVNEVTEFAK** | **66-75** | **575.31++** | **937.46+** | **9.4** | **389,185** |  |
|  | **SLHTLFGDK** | **89-97** | **339.85+++** | **466.23+** | **6.4** | **208,593** |  |
|  | **DDNPNLPR** | **131-138** | **470.73++** | **596.35+** | **3.4** | **311,779** |  |
|  | **YLYEIAR** | **162-168** | **464.25++** | **651.35+** | **8.2** | **917,563** |  |
|  | AEFAEVSK | 250-257 | 440.72++ | 680.36+ | 3.4 | 206,341 |  |
|  | **LVTDLTK** | **258-264** | **395.24++** | **577.32+** | **4.7** | **934,495** |  |
|  | **TYETTLEK** | **376-383** | **492.75++** | **720.38+** | **3.3** | **43,248** |  |
|  | VFDEFKPLVEEPQNLIK | 397-413 | 682.37+++ | 899.98++ | 17.7 | 395,646 |  |
|  | **FQNALLVR** | **427-434** | **480.78++** | **685.44+** | **8.8** | **729,918** |  |
|  | VPQVSTPTLVEVSR | 439-452 | 756.43++ | 1088.59+ | 11.7 | 42,875 |  |
|  | **QTALVELVK** | **550-558** | **500.81++** | **587.38+** | **10.3** | **385,910** |  |
|  | AVMDDFAAFVEK | 570-581 | 671.82++ | 1041.49+ | 17.5 | 51,445 |  |
| Skyline | LVNEVTEFAK | 66-75 | 575.31++ | 595.31+ | 9.5 | 159,825 |  |
|  | TCVADESAENCDK | 76-88 | 749.79+ | 1067.40+ | 1.4 | 806 |  |
|  | SLHTLFGDK | 89-97 | 509.27++ | 680.36+ | 6.4 | 38,014 |  |
|  | **LCTVATLR** | **98-105** | **467.26++** | **660.40+** | **5.9** | **638,353** |  |
|  | ETYGEMADCCAK | 106-117 | 771.77++ | 855.32+ | 4.5 | 1,198 |  |
|  | NECFLQHK | 123-130 | 538.25++ | 672.38+ | 2.2 | 7,115 |  |
|  | DDNPNLPR | 131-138 | 470.73++ | 596.35+ | 3.3 | 178,252 |  |
|  | HPYFYAPELLFFAK | 170-183 | 871.95++ | 964.55+ | 20.9 | 2,205 |  |
|  | AAFTECCQAADK | 187-198 | 686.29++ | 981.38+ | 3.3 | 5,500 |  |
|  | AEFAEVSK | 250-257 | 440.72++ | 680.36+ | 3.4 | 269,595 |  |
|  | VHTECCHGDLLECADDR | 265-281 | 1043.92++ | 1163.50+ | N/A | N/A |  |
|  | YICENQDSISSK | 287-298 | 722.32++ | 878.42+ | 3.9 | 3,265 |  |
|  | DVFLGMFLYEYAR | 348-360 | 812.40++ | 961.48+ | 25.0 | 12,647 |  |
|  | HPDYSVVLLLR | 362-372 | 656.37++ | 1174.89+ | 14.2 | 8,022 |  |
|  | TYETTLEK | 376-383 | 492.75++ | 720.38+ | 3.3 | 51,773 |  |
|  | CCAAADPHECYAK | 384-396 | 776.80++ | 904.40+ | N/A | N/A |  |
|  | VFDEFKPLVEEPQNLIK | 397-413 | 1023.05+++ | 1279.76+ | 17.7 | 5,473 |  |
|  | FQNALLVR | 427-434 | 480.78++ | 685.44+ | 8.8 | 788,438 |  |
|  | VPQVSTPTLVEVSR | 439-452 | 756.43++ | 900.51+ | 11.7 | 15,069 |  |
|  | CCTESLVNR | 500-508 | 569.75++ | 818.44+ | 3.2 | 13,103 |  |
|  | RPCFSALEVDETYVPK | 509-524 | 955.97++ | 1263.65+ | N/A | N/A |  |
|  | QTALVELVK | 550-558 | 500.81++ | 587.38+ | 10.3 | 241,180 |  |
|  | AVMDDFAAFVEK | 570-581 | 671.82++ | 1041.49+ | 17.4 | 30,831 |  |
|  | ETCFAEEGK | 589-597 | 535.73++ | 941.40+ | N/A | N/A |  |

**Table S6.** Hydrophobicity indexes and predicted water solubility of signature and SIL extended peptides ordered by increasing hydrophobicity.

| **Type of peptide or SIL-Ex** | **Surrogate peptide sequence** | **Hydrophobicity** | **Predicted water solubility** |
| --- | --- | --- | --- |
| SIL-TCT | DLGEENFKSAnYG | --- | good |
| SIL-TCT | LVNEVTEFAKSAnYG | --- | good |
| SIL-TCT | SLHTLFGDKSAnYG | --- | good |
| SIL-TCT | LCTVATLRSAnYG | --- | poor |
| SIL-TCT | DDNPNLPRSAnYG | --- | good |
| SIL-TCT | YLYEIARSAnYG | --- | poor |
| SIL-TCT | LVTDLTKSAnYG | --- | good |
| SIL-TCT | TYETTLEKSAnYG | --- | good |
| SIL-TCT | FQNALLVRSAnYG | --- | poor |
| SIL-TCT | QTALVELVKSAnYG | --- | good |
| signature peptide | DDNPNLPR | 11.99 | good |
| signature peptide | TYETTLEK | 13.58 | good |
| signature peptide | DLGEENFK | 16.44 | good |
| signature peptide | LVTDLTK | 17.02 | good |
| SIL-TCEx5 | TYETTLEKCCAAA | 18.19 | good |
| signature peptide | LCTVATLR | 20.29 | good |
| SIL-TCEx5 | LVTDLTKVHTEC | 21.02 | good |
| SIL-TCEx3_3 | LQHKDDNPNLPRLVR | 22.59 | good |
| signature peptide | SLHTLFGDK | 23.32 | good |
| SIL-TCEx5 | DDNPNLPRLVRPE | 24.05 | good |
| signature peptide | YLYEIAR | 24.44 | good |
| signature peptide | LVNEVTEFAK | 24.51 | good |
| SIL-TCEx5 | LCTVATLRETYGE | 24.82 | good |
| signature peptide | FQNALLVR | 27.03 | good |
| signature peptide | QTALVELVK | 27.44 | good |
| SIL-TCEx3_3 | LKKYLYEIARRHP | 28.31 | good |
| SIL-TCEx5 | QTALVELVKHKPKA | 28.39 | good |
| SIL-TCEx5 | LVNEVTEFAKTCVAD | 28.59 | good |
| SIL-TCEx3_3 | CDKSLHTLFGDKLCT | 28.82 | good |
| SIL-TCEx3_3 | IKKQTALVELVKHKP | 29.26 | good |
| SIL-TCEx5_5 | FLQHKDDNPNLPRLVRPE | 30.42 | good |
| SIL-TCEx5_5 | RQIKKQTALVELVKHKPKA | 30.84 | good |
| SIL-TCEx5 | FQNALLVRYTKKV | 31.14 | good |
| SIL-TCEx5 | SLHTLFGDKLCTVA | 32.56 | poor |
| SIL-TCEx5_5 | AENCDKSLHTLFGDKLCTVA | 32.81 | good |
| SIL-TCEx5 | YLYEIARRHPYF | 34.30 | poor |
| SIL-TCEx3_3 | HVKLVNEVTEFAKTCV | 35.04 | good |
| SIL-TCEx5 | DLGEENFKALVLI | 37.59 | good |
| SIL-TCEx5_5 | FEDHVKLVNEVTEFAKTCVAD | 38.75 | good |
| SIL-TCEx5_5 | TFLKKYLYEIARRHPYF | 45.42 | good |

*^a^* Hydrophobicity was calculated using Thermo Fisher Peptide Analyzing Tool. To predict water solubility, the Innovagen Peptide solubility calculator was used.

**Table S7.** SIL-TCT and SIL-ExC5 peptides' quantitative performance reconstituted in water with 5% ACN and AmBic/SDC buffer with 5% ACN.

| **Surrogate peptide sequence** | **SIL peptides reconstituted in 5% ACN in water** | | | | | | **SIL peptides reconstituted in 5% ACN in AmBic/SDC** | | | | | |
| --- | --- | --- | --- | --- | --- | --- | --- | --- | --- | --- | --- | --- |
|  | **SIL-TCT** | | | **SIL-TCEx5** | | | **SIL-TCT** | | | **SIL-TCEx5** | | |
|  | **nM** | **CV between triplicates (%)** | **CV from actual HSA concentration (%)** | **nM** | **CV between triplicates (%)** | **CV from actual HSA concentration (%)** | **nM** | **CV between triplicates (%)** | **CV from actual HSA concentration (%)** | **nM** | **CV between triplicates (%)** | **CV from actual HSA concentration (%)** |
| **DLGEENFK** | 42.53 | 4.52 | 91.49 | 74.88 | 19.96 | 85.02 | 7.40 | 7.38 | 98.52 | 5.42 | 11.73 | 98.92 |
| **LVNEVTEFAK** | 1,164.73 | 13.36 | 132.95 | 689.85 | 16.24 | 37.97 | 343.12 | 2.07 | 31.38 | 234.24 | 11.66 | 53.15 |
| **SLHTLFGDK** | 1,190.16 | 8.80 | 138.03 | 880.75 | 18.16 | 76.15 | 188.88 | 10.21 | 62.22 | 361.20 | 8.52 | 27.76 |
| **LCTVATLR** | 708.69 | 3.27 | 41.74 | 473.78 | 11.16 | 5.24 | 175.44 | 12.00 | 64.91 | 126.98 | 13.29 | 74.60 |
| **DDNPNLPR** | 188.86 | 1.69 | 62.23 | 163.67 | 3.28 | 67.27 | 82.50 | 14.05 | 83.50 | 71.61 | 3.41 | 85.68 |
| **YLYEIAR** | 1,009.32 | 13.88 | 101.86 | 1,501.44 | 11.86 | 200.29 | 382.39 | 2.33 | 23.52 | 383.26 | 3.87 | 23.35 |
| **LVTDLTK** | 542.71 | 7.48 | 8.54 | 455.96 | 4.33 | 8.81 | 363.22 | 8.12 | 27.36 | 278.47 | 2.59 | 44.31 |
| **TYETTLEK** | 435.29 | 1.55 | 12.94 | 326.55 | 2.35 | 34.69 | 288.15 | 3.75 | 42.37 | 295.39 | 4.35 | 40.92 |
| **FQNALLVR** | 1,723.73 | 12.40 | 244.75 | 1,384.51 | 13.95 | 176.90 | 369.81 | 2.05 | 26.04 | 656.61 | 6.54 | 31.32 |
| **QTALVELVK** | 1,670.96 | 10.47 | 234.19 | 715.89 | 6.77 | 43.18 | 345.95 | 5.45 | 30.81 | 320.92 | 4.59 | 35.82 |
| **Average** | **867.70** | **7.74** | **106.87** | **666.73** | **10.81** | **73.55** | **254.69** | **6.74** | **49.06** | **273.41** | **7.05** | **51.58** |

**Table S8.** SPE recoveries of light serum peptides, SIL-HSA, and heavy/light peptide ratio.

| **Surrogate peptide sequence** | **Recovery (%)** | | **SIL-HSA recovery/light HSA recovery** |
| --- | --- | --- | --- |
|  | **Light HSA** | **SIL-HSA** |  |
| **DLGEENFK** | 70.03 | 70.80 | 1.01 |
| **LVNEVTEFAK** | 73.98 | 73.35 | 0.99 |
| **SLHTLFGDK** | 73.28 | 68.85 | 0.94 |
| **LCTVATLR** | 70.65 | 66.54 | 0.94 |
| **DDNPNLPR** | 81.34 | 76.54 | 0.94 |
| **YLYEIAR** | 71.52 | 73.60 | 1.03 |
| **LVTDLTK** | 76.82 | 70.08 | 0.91 |
| **TYETTLEK** | 71.90 | 65.70 | 0.91 |
| **FQNALLVR** | 62.91 | 60.19 | 0.96 |
| **QTALVELVK** | 71.08 | 73.19 | 1.03 |
